# Identification of a putative quantitative trait nucleotide in guanylate binding protein 5 for host response to PRRS virus infection

**DOI:** 10.1186/s12864-015-1635-9

**Published:** 2015-05-28

**Authors:** James E. Koltes, Eric Fritz-Waters, Chris J. Eisley, Igseo Choi, Hua Bao, Arun Kommadath, Nick V. L. Serão, Nicholas J. Boddicker, Sam M. Abrams, Martine Schroyen, Hyelee Loyd, Chris K. Tuggle, Graham S. Plastow, Leluo Guan, Paul Stothard, Joan K. Lunney, Peng Liu, Susan Carpenter, Robert R. R. Rowland, Jack C. M. Dekkers, James M. Reecy

**Affiliations:** Department of Animal Science, Iowa State University, 2255 Kildee Hall, Ames, IA 50011 USA; Department of Statistics, Iowa State University, 1121 Snedecor Hall, Ames, IA 50011 USA; USDA-ARS, BARC, APDL, Building1040, Beltsville, MD 20705 USA; Department of Agricultural, Food and Nutritional Science, University of Alberta, Edmonton, AB T6G 2P5 Canada; Genesus Inc, 101 2nd Street, Oakville, MB R0H 0Y0 Canada; College of Veterinary Medicine, Kansas State University, K-231 Mosier Hall, Manhattan, KS 66506 USA

## Abstract

**Background:**

Previously, we identified a major quantitative trait locus (QTL) for host response to Porcine Respiratory and Reproductive Syndrome virus (PRRSV) infection in high linkage disequilibrium (LD) with SNP rs80800372 on *Sus scrofa* chromosome 4 (SSC4).

**Results:**

Within this QTL, guanylate binding protein 5 (*GBP5*) was differentially expressed (DE) (p < 0.05) in blood from AA versus AB rs80800372 genotyped pigs at 7,11, and 14 days post PRRSV infection. All variants within the *GBP5* transcript in LD with rs80800372 exhibited allele specific expression (ASE) in AB individuals (p < 0.0001). A transcript re-assembly revealed three alternatively spliced transcripts for *GBP5*. An intronic SNP in *GBP5*, rs340943904, introduces a splice acceptor site that inserts five nucleotides into the transcript. Individuals homozygous for the unfavorable AA genotype predominantly produced this transcript, with a shifted reading frame and early stop codon that truncates the 88 C-terminal amino acids of the protein. RNA-seq analysis confirmed this SNP was associated with differential splicing by QTL genotype (p < 0.0001) and this was validated by quantitative capillary electrophoresis (p < 0.0001). The wild-type transcript was expressed at a higher level in AB versus AA individuals, whereas the five-nucleotide insertion transcript was the dominant form in AA individuals. Splicing and ASE results are consistent with the observed dominant nature of the favorable QTL allele. The rs340943904 SNP was also 100 % concordant with rs80800372 in a validation population that possessed an alternate form of the favorable B QTL haplotype.

**Conclusions:**

GBP5 is known to play a role in inflammasome assembly during immune response. However, the role of GBP5 host genetic variation in viral immunity is novel. These findings demonstrate that rs340943904 is a strong candidate causal mutation for the SSC4 QTL that controls variation in host response to PRRSV.

**Electronic supplementary material:**

The online version of this article (doi:10.1186/s12864-015-1635-9) contains supplementary material, which is available to authorized users.

## Background

Recently, Boddicker et al. [1, 2] identified a major QTL on *Sus scrofa* chromosome 4 (SSC4) at 139136697–140420778 that accounts for 15.7 % and 11.2 % of the genetic variance for host response of young pigs to experimental PRRS virus infection, as measured by serum viremia from 0 to 21 days post infection (dpi) and weight gain to 42 dpi. In addition, a SNP (rs80800372) was identified that appeared to be in complete LD with the QTL. Biological candidate genes in the region that have been previously studied include members of the guanylate binding protein (GBP) family [3]. The five members of the GBP family that are located in the QTL region all represent potential candidate genes for the QTL effect. The GBP proteins work together in an interferon (IFN)-inducible complex to mediate a proinflammatory immune response to stimuli such as the gram-negative bacterial cell wall component lipopolysaccharide (LPS) [4]. The GBPs can stimulate caspase-11-dependent pyroptosis, a specific form of apoptosis triggered by pathological stimuli such as contact with microbes, toxins or viruses [5]. Both GBP2 and GBP3 have been associated with the ability to control pathogen replication [3, 6] but *GBP3* is not assigned to the SSC4 QTL region. The GBP5 protein has previously been shown to play a role in immune response through mediation of inflammasome assembly [7]. Thus, the SSC4 QTL region includes several very good biological candidate genes, but because of high LD in the region, further fine mapping of the QTL is problematic.

The GBP5 protein is an important mediator of inflammatory immune response in mammals. Loss of *GBP5* function in a knockout mouse model results in impaired host defense and inflammatory response because GBP5 facilitates nucleotide binding and oligomerization, leucine-rich repeat protein 3 (NLRP3) mediated inflammasome assembly [7]. Specifically, NLRP3 interacts with tetrameric GBP5 to promote inflammasome assembly with apoptosis-associated speck-like protein containing a caspase activation and recruitment domain protein (ASC) [7]. The 62 C-terminal amino acids of GBP5 are required for tetramerization, and loss of tetrameric GBP5 abolished NLRP3 inflammasome mediated ASC assembly [7]. Mice lacking functional GBP5 had significantly reduced neutrophil recruitment in response to peritonitis. Further, these knockout mice also had increased bacterial burdens, severely reduced CD11b + cells in mesenteric lymph nodes and noticable weight loss in response to *L. monocytogenes* infection [7]. In addition, one study indicated that *GBP5* expression was upregulated in response to Epstein-Barr viral infection [8]. However, the role and connection between GBP5 and viral immune response has not been characterized.

The objective of this study was to use functional genomics methods to identify the gene and mutation responsible for the observed QTL on SSC4 for host response to PRRS virus infection. We present evidence for a putative causal mutation based on differential expression, allele specific expression and differential splicing data that created an illegitimate splice site in *GBP5*. Furthermore, we show that the mutation is in 100 % linkage disequilibrium (LD) with the previously reported SSC4 QTL in an independent population.

## Results

### De novo transcript assembly to improve gene expression quantification in the SSC4 region

Several genomic assembly issues were identified in the SSC4 QTL region that made accurate quantification of gene expression problematic. For example, the *GBP5* transcript in the *Sus scrofa* 10.2 genome assembly (Sscrofa build 10.2) was missing the C-terminal end of *GBP5* that contains a conserved CAAX box domain required for cellular localization. Using translated BLAST to compare RNA-seq reads to the human protein, exon 11 of *GBP5* was identified approximately 200 kb 5′ of the start site of *GBP5* in Sscrofa build 10.2. In addition, the predicted Ensembl gene *ENSSSCG00000027014* was noted to have nearly equal identity to fragments of *GBP2* and *GBP4*. We also identified several gaps in the reference sequence within the QTL region in Sscrofa build10.2 that were indicative of assembly problems. A transcript reassembly was conducted, after which all transcripts were compared to the pig and human genomes to ensure the presence of the entire protein coding sequence. For reference, the original order of the genes from the Sscrofa build 10.2 is provided in Additional file 1. Full-length transcripts were identified for *GBP4*, *GBP5*, *GBP6*, *GTF2B*, *CCBL2*, and *PKN2*. The *GBP1* and *GBP2* transcripts were assembled utilizing the existing transcript information and the human transcripts for these genes. The FASTA formatted sequence of all cDNAs corresponding to the eight genes assembled is provided in Additional file 2. We were unable to identify fragments of the *ENSSSCG00000027014* transcript from Sscrofa build 10.2 in our de -novo assembly. Thus, this gene may be the product of a genome misassembly, may not be expressed in whole blood, or may be a non-functional gene duplication. The syntenic human chromosomal block to the QTL region includes the *GBP3* gene but this gene was not found in the de novo transcriptome assembly or by BLAST search of the human GBP3 protein against either the RNA-seq reads or our de novo transcript assembly of this region. This gene is also missing from Sscrofa build 10.2.

### Read mapping statistics

Seventy sample sequences from 16 pigs and 5 time points following experimental infection were retained after quality control for identification of differentially expressed (DE) genes (Additional file 3). The average percentage of reads that uniquely mapped to the Sscrofa build10.2 reference genome for these 70 samples was 82.1 %. Mapping percentages for individual samples are provided in Additional file 4.

### Identification of differentially expressed genes due to QTL genotype

**Fig. 1:**
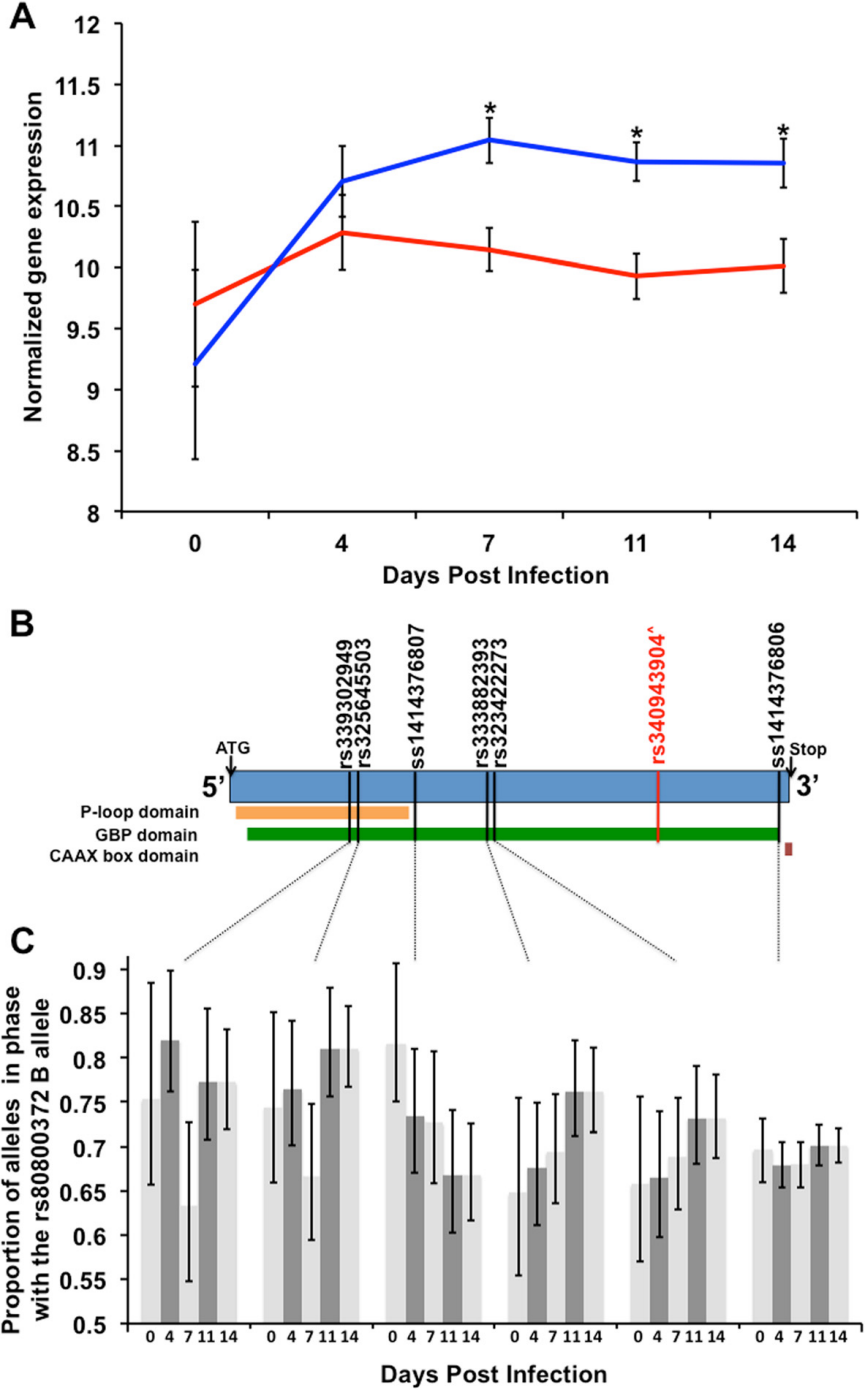
GBP5 is differentially expressed as a function of the PRRS host response QTL across timepoints. Guanylate binding protein 5 (*GBP5*) is differentially expressed and exhibits allele specific expression (ASE) as a function of rs80800372 genotype at 0,4,7,11 and 14 days post infection (dpi) with Porcine Reproductive and Respiratory Syndrome (PRRS) in PHGC trial 3 RNA-seq data. **a** Differences in the mean, model normalized gene expression value and log2 fold change values for expression of *GBP5* between the AB (blue) and AA (red) genotypes at the SSC4 QTL. *Indicates false discovery rate < 0.05. **b** Schematic of the *GBP5* transcript coding DNA sequence. Variants tested for ASE are shown in black text. The putative causal mutation is shown in red text. The three protein domains shown were identified from the human GBP5 protein as annotated at Ensembl in release 77. The box shown in orange corresponds to the p-loop containing nucleoside triphosphate hydrolase domain (7–279 amino acids (AA)). The box shown in green corresponds to the GBP N and C terminal domains (18–280 AA and 282–577 AA, respectively). The box shown in red corresponds to the CAAX box localization signal required for proper cellular localization of the GBP5 protein. ^^^The rs340943904 variant is not included in the transcript and is shown here only to provide context since it is predicted to cause a shift in the transcript reading frame and an early stop codon that truncates the 88 C-terminal AA of the protein. **c** Identification of ASE at 0,4,7,11 and 14 dpi for *GBP5* for SNPs in phase with the B rs80800372 allele. The 95 % confidence intervals for estimates of allele proportions are provided

All eight gene transcripts from the SSC4 QTL region were analyzed for differential expression, with a primary focus on the effects of QTL genotype and genotype by dpi. A total of three transcripts were DE due to genotype across dpi, and 1, 3, and 1 transcripts were DE due to genotype by day effects on 7, 10 and 14 dpi, respectively (p < 0.05). No transcripts were DE at 0 and 4 dpi. Significant transcript log2 fold changes are provided in Fig. 1a. Genes that were DE due to genotype included *GBP4*, *GBP5*, and *GBP6*; however, only *GBP5* was DE across multiple time points (7, 10 and 14 dpi) (Fig. 1a). The log2 fold changes, p-values and false discovery rates (FDR) for all genes in the SSC4 QTL region are presented in Additional files 5, 6, and 7, respectively.

### Allele Specific Expression (ASE) analysis identified higher expression of *GBP5* alleles in LD with the favorable QTL allele

A total of 64 SNPs in the eight genes in the SSC4 QTL region were in complete LD with the SSC4 QTL based on concordance with rs80800372 genotype. These 64 SNPs were analyzed for ASE in animals that were heterozygous for rs80800372. All SNPs were filtered by minimum read depth to avoid the analysis of false SNPs due to sequencing errors. Of these 64 SNPs, 43 SNPs exhibited ASE when averaged across dpi while FDR was controlled at 5 %. The greatest number of SNPs identified that exhibited ASE per gene were in *GBP2* (9 SNPs), *GBP4* (9 SNPs), *GBP6* (7 SNPs), and *GBP5* (6 SNPs). The estimated probability of expression of the alternate allele (p_alt_) averaged across all dpi time points for these SNPs in LD with the QTL are presented in Additional file 8. However, *GBP5* was the only gene with all SNPs in LD that showed consistent main effects for ASE across all SNPs tested, as presented in Fig. 1b and c. Estimates of allelic proportions and p-values for these ASE SNPs are presented for each dpi in Additional file 8. A list of ASE results for additional SNPs that were not in complete LD with rs80800372 is provided in Additional file 9.

### Identification of a putative causal mutation in *GBP5* that introduces an illegitimate splice acceptor site

**Fig. 2:**
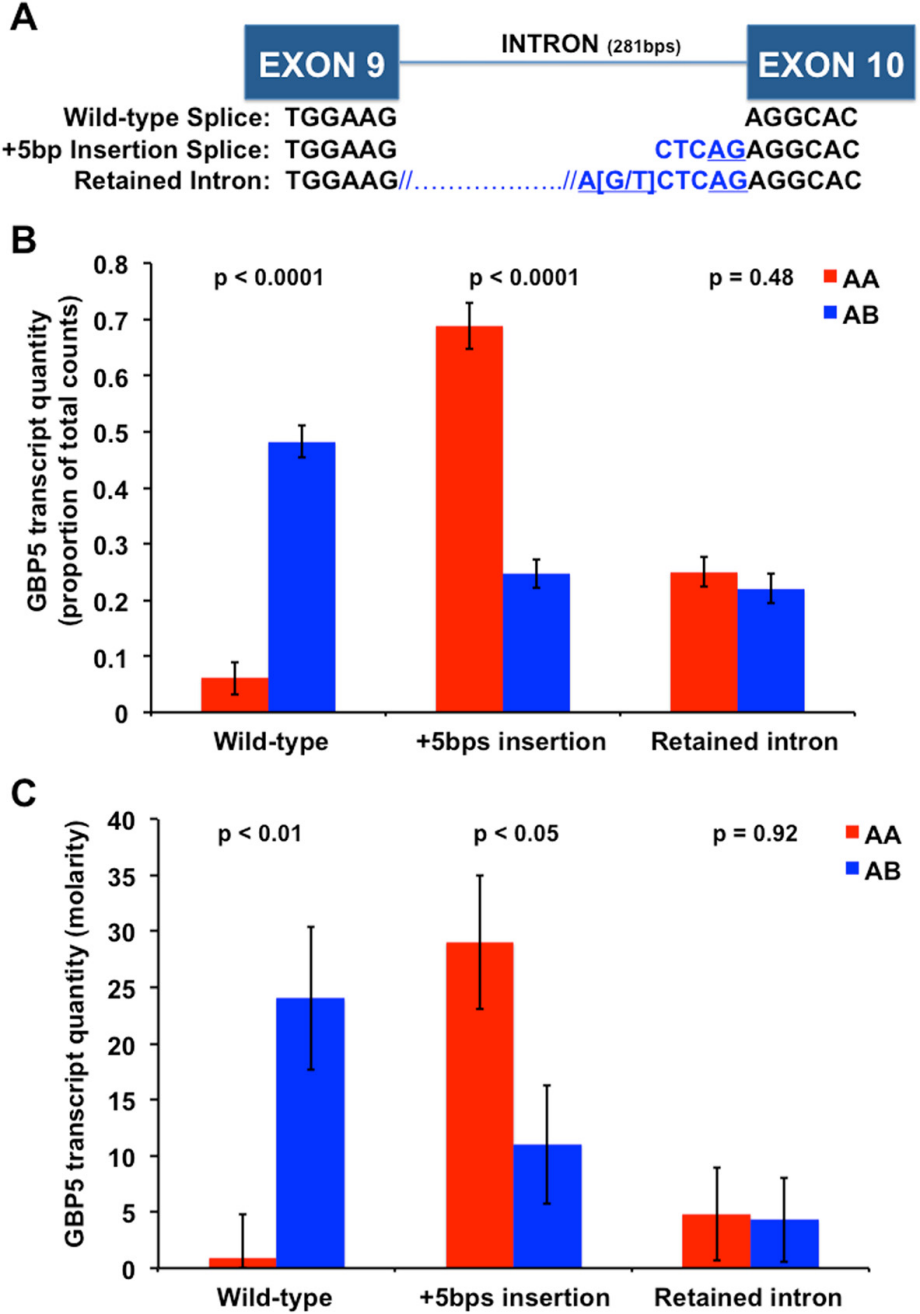
Association and validation of guanylate binding protein 5 (*GBP5*) differential splicing with rs80800372 genotype. **a** Schematic of the sequence level differences between the three alternate transcripts of *GBP5*, including the position of the putative causal variant (rs340943904, bracketed). The schematic displays how the rs340943904 G allele creates a new splice acceptor site in intron 9 (underlined blue text) while the T allele destroys the splice site. The wild-type splicing transcript includes only the sequence in black, while the +5bp insert transcript includes the extra five nucleotides shown in blue. The retained intron transcript includes the entire 281 nucleotide intron. The rs340943904 G allele is in perfect linkage disequilibrium (LD) with the unfavorable rs80800372 “A” allele while the rs340943904 T allele is in perfect LD with the favorable rs80800372 “B” allele. **b** Differential splicing of *GBP5* by rs80800372 genotype across all days post infection (dpi) time points. Splicing was measured as the percentage of read counts from each alternate transcript standardized for all read counts from the PHGC trial 3 RNA-seq data. This figure demonstrates that very little wild-type splicing transcript is produced by the AA (red) genotype compared to the AB (blue) rs80800372 genotype (p<0.0001; Bonferonni adjusted p<0.001). In contrast, considerably more of the alternate transcript (+5bps insertion) is produced in the AA compared to the AB rs80800372 genotyped individuals. **c** Confirmation of *GBP5* differential splicing due to rs80800372 genotype across all dpi following infection using quantitative capillary electrophoresis. This analysis validates that very little or no wild-type splicing transcript is produced by AA (red) vs. AB (blue) SSC4 rs80800372 genotyped individuals (p<0.01; Bonferonni adjusted p<0.05). Consistent with the RNA-seq results, more of the alternate transcript with five extra nucleotides is quantified in blood RNA from AA vs. AB rs80800372 genotyped individuals. Expression values are presented as transcript molarity

Since *GBP5* was DE between QTL genotypes at several dpi, exhibited ASE, and was a strong biological candidate gene, we carefully examined the RNA sequence data of this gene for variants. Interestingly, a T > G SNP (sense strand) was identified in intron 9 of *GBP5* that appeared to introduce an additional splice acceptor site five base pairs 5′ to the start of exon 10 in animals that possessed the unfavorable A QTL allele. This splice acceptor SNP was previously assigned the NCBI dbSNP ID rs340943904. The position of rs340943904 relative to SNPs exhibiting ASE and the gene structure of *GBP5* is presented in Fig. 2a. The context of rs340943904 within the *GBP5* transcript is presented in Additional file 10. Reads that mapped to *GBP5* exon 9, intron 9 and exon 10 were evaluated for splicing consistent with multiple alternatively spliced transcripts (Additional file 11). Three alternate transcripts were identified, including the wild-type transcript, a transcript with a five nucleotide insertion 5′ to exon 10 (+5 bps transcript), and a transcript that retained intron 9 (retained intron transcript). The rs340943904 G allele introduces a new splice acceptor site, which causes five nucleotides to be inserted into the GBP5 transcript. This five nucleotide insertion is expected to shift the reading frame of GBP5, altering the first 10 amino acids encoded by exon 10 and introduces an early stop codon that truncates the 88 C-terminal amino acids of the protein, including the CAAX box. Transcripts that contained the five-nucleotide insertion were identified consistently in individuals with the AA QTL genotype, but had a much lower frequency in individuals with the AB QTL genotype. The transcript assembly confirmed the presence of three *GBP5* alternatively spliced transcripts (wild-type, +5 bps and retained intron). The sequence of the wild-type transcript is provided in Additional file 12. Additional file 13 shows the predicted frameshift due to the rs340943904 G allele at the protein level in AA and AB QTL genotype individuals in comparison to the amino acid sequence of GBP5 in human, cattle and mouse using CLUSTALW [9]. The predicted effects of all other variants detected in linkage disequilibrium or equilibrium with rs80800372 are listed in Additional files 14 and 15, respectively.

### *GBP5* exhibited differential splicing

To evaluate the relationship of altered *GBP5* splicing with the SSC4 QTL genotype, all RNA-seq reads that discriminate the three alternatively spliced transcripts were counted. Altered splicing was observed to segregate consistently with the AA and AB rs80800372 genotypes. A significantly lower number of wild-type splicing read counts was present in AA compared to AB individuals, after standardization of each alternate transcript as the proportion of total reads produced per sample across all three transcripts (p < 0.0001; Fig. 2b).

### Independent validation of *GBP5* differential splicing

The correspondence of differential splicing to the PRRS QTL genotype was further validated in an analysis across five dpi using AATI capillary electrophoresis technology (Fig. 2c). The wild-type splicing transcript was identified as over-expressed in RNA from whole blood of AB vs. AA genotype animals (p < 0.01). In contrast, the +5 bp insertion allele was identified as over-expressed in the AA vs. AB genotype animals (p < 0.05). Abundance of the retained intron appeared to be similar between genotypes. Removal or inclusion of the retained intron in the statistical analysis did not change the observed results.

### RNA-seq validation using an independent population

**Fig. 3:**
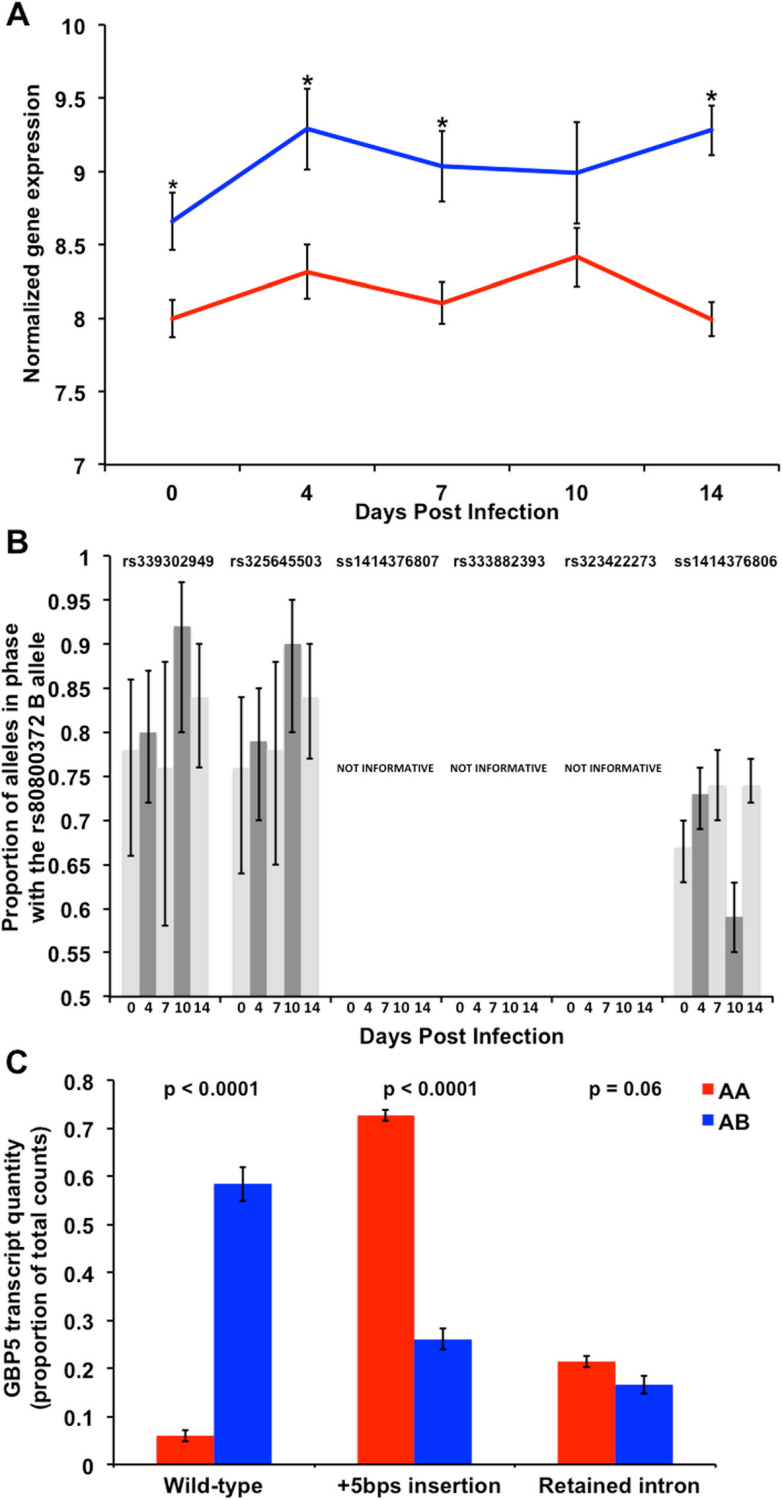
Validation of differential expression, splicing and allele specific expression of guanylate binding protein 5 (*GBP5*). *GBP5* transcript levels and allelic abundances were analyzed at 0,4,7,10 and 14 days post infection (dpi) with Porcine Reproductive and Respiratory Syndrome (PRRS) virus in PHGC trial 5 RNA-seq data. **a** Validation of differential expression of *GBP5* between the AB (blue) and AA (red) rs80800372 genotypes. *Indicates false discovery rate < 0.05. **b** Validation of allele specific expression of *GBP5* SNPs in phase with the B rs80800372 allele. **c** Validation of *GBP5* differential splicing due to rs80800372 genotype across dpi. Spliced reads uniquely mapping to the three alternate transcripts were analyzed as the percentage of total reads from the RNA-seq data. Both the wild-type and five nucleotide insertion (+5 bps insertion) alleles were differentially spliced between the AA (red bars) and AB (blue bars) rs80800372 genotypes (p < 0.0001; Bonferonni adjusted p < 0.001). There was a trend towards a small difference in the quantity of the retained intron due to rs80800372 genotype (p = 0.06; Bonferonni adjusted p > 0.10). Alternate *GBP5* transcripts are labeled on the X-axis

Gene expression data from animals of a different genetic background used in an independent validation study were used to determine if *GBP5* was DE between the PRRS QTL genotypes. In the validation population (PRRS Host Genome Consortium trial 5 (PHGC5)), *GBP5* expression was measured by RNA-seq and analyzed in AA and AB rs80800372 genotyped pigs at the same five time points (0,4,7,10 and 14 dpi; Fig. 3a) following experimental PRRS infection as in the present study. *GBP5* was DE for the main effect of genotype and at four of the five dpi time points (FDR < 0.05; Fig. 3a). Consistent with the first study, *GBP5* differential splicing was associated with the rs340943904 SNP genotype in the validation population (p < 0.0001; Fig. 3c). Three SNPs within the *GBP5* transcript were identified to exhibit ASE (Fig. 3b). However, three SNPs (ss1414376807, rs333882393, rs323422273) did not exhibit ASE. At least 3 of the 6 AB individuals with RNA-seq data appear to be homozygous, and likely uninformative. These results validate that *GBP5* RNA levels, splicing and ASE after PRRSV infection are different between PRRS SSC4 QTL genotypes and that the rs340943904 SNP is the putative causative mutation through alternative splicing.

### Concordance of the *GBP5* variant rs340943904 with rs80800372 in an independent population

An allele specific SNP assay was developed to genotype both the *GBP5* rs340943904 and rs80800372 variants. A total of 58 boars from an independent commercial Yorkshire breeding population were genotyped to determine the correspondence of the putative causal variant in *GBP5* with the rs80800372 SNP that tracks the PRRS QTL. Importantly, despite the presence of two different B rs80800372 QTL haplotypes [2], one in PHGC3 and a second in the PHGC5 population, the rs340943904 SNP is 100 % concordant (data not shown).

## Discussion

The goal of this study was to identify the gene and causal variant responsible for the SSC4 QTL for PRRS resistance. A secondary goal was to identify potential molecular mechanisms for the QTL. The results of the RNA-seq study provide multiple lines of evidence that the rs340943904 splice acceptor mutation in *GBP5* is a strong candidate causal mutation for this QTL.

Porcine reproductive and respiratory syndrome (PRRS) is the most costly infectious disease in the global pig industry [10] with an annual cost of $664 million to the US industry alone [11]. PRRS is caused by infection with an enveloped positive stranded RNA virus [12, 13]. Clinical signs of PRRS include late-term reproductive failure in sows and respiratory illness in growing pigs (reviewed in [14]). PRRS virus is also a cofactor in other disease syndromes, such as porcine respiratory disease complex (PRDC) and porcine circovirus-associated disease (PCVAD) [15, 16]. Vaccination for protection against PRRSV infection has generally been unsuccessful, primarily due to the high degree of antigenic and genetic drift in viral structural and non-structural proteins and the capacity of the virus to subvert early innate immune responses [17]. Genetics of the host has been well documented as another factor associated with susceptibility and response of the pig to PRRS [18–20]. The impact of PRRS on swine health, well-being and severity of additional pathogen infections when combined with PRRS underscores the importance of host genetics and major gene variation in porcine immune response.

Three genes were DE between the AA (unfavorable) and AB (favorable) PRRS host response QTL genotypes (FDR < 0.05). However, only *GBP5* was identified as DE across multiple time points post infection (FDR < 0.05) and across all time points (FDR = 0.051). This result was validated in an independent RNA-seq experiment from a different pig population obtained through the PRRS host genetics consortium (PHGC). Since this second study was not designed specifically to identify differences in gene expression due to the SSC4 QTL genotype, but also focused on the effects of different host response phenotypes, this result provides strong evidence that *GBP5* is DE due to QTL genotype. This result indicates that differential expression of *GBP5* is related to or mediates the QTL effect on PRRS host response.

To provide additional evidence for differences in expression, an ASE analysis of all SNPs in complete LD with the PRRS host response QTL was conducted to determine if evidence of allelic imbalance exists in correspondence with the QTL genotype. *GBP5* was the only gene where all SNPs in LD with QTL exhibited ASE. This result was validated in the independent PHGC5 RNA-seq data set for three of the six SNPs. The three SNPs that were not ASE appeared to be uninformative based on the lack of expression of an alternate allele in half of the AB animals in the PHGC5 dataset, which is supported by the multiple B QTL haplotypes identified in this population [2]. There are many potential reasons for statistical evidence of ASE, including differences in regulatory factor binding, imprinting, DNA methylation, RNA splicing, RNA stability, RNA editing, and miRNA mediated silencing. Since all SNPs in *GBP5* exhibited ASE, we hypothesized that ASE in *GBP5* may occur due to differences in RNA stability or splicing.

Evidence from RNA-seq data and de novo assembly indicated that *GBP5* exhibited alternative splicing. Concurrently, we identified the rs340943904 SNP in *GBP5*, for which the allele that was in perfect LD with the unfavorable A allele at the SSC4 QTL inserted five nucleotides into the *GBP5* transcript upstream of exon 10. This insertion is expected to result in an early stop codon and thus a non-functional GBP5 protein. Since this SNP was predicted to change the splicing of *GBP5*, we used RNA-seq reads spanning the specific splice junctions to quantify the three alternatively spliced *GBP5* transcripts across all time points. The wild-type splicing transcript was expressed more in individuals with the AB QTL genotype (p < 0.001), while the five nucleotide insertion transcript was more highly expressed in AA individuals (p < 0.001). No difference in expression was observed for the retained intron transcript between genotypes. Splicing consistent with these results was observed in a second population (Fig. 2c). We validated the differential splicing results using quantitative capillary electrophoresis (p < 0.01). Interestingly, we could not detect the wild-type splicing transcript in AA individuals using this method. These results indicate that individuals with the AA QTL genotype produce almost no wild-type *GBP5* transcript and an excess of the transcript that is expected to produce a non-functional protein. Thus, we predict that individuals with the AA QTL genotype produce very little functional GBP5 protein. None of the other 63 SNPs in LD identified in the QTL region (Additional files 12 and 13). The facts that AB QTL genotyped individuals produce significantly more wild-type splicing transcript than AA individuals, and that AA individuals produce almost no wild-type splicing *GBP5*, while producing large amounts of a non-functional transcript, are consistent with the observed dominance of the B QTL allele [21]. It is unclear whether the differential expression of *GBP5* completely depends on differential splicing, since the rs340943904 SNP is in perfect LD with the PRRS host response QTL. Thus, it is difficult to determine whether the genotype-specific differences in *GBP5* expression are directly related to changes in splicing or caused by other mechanisms involved in transcript stability such as nonsense mediated decay.

To further validate the degree of concordance of the rs340943904 SNP with the SSC4 PRRS host response QTL, this SNP was genotyped along with the rs80800372 SNP in an independent set of pigs from a different PHGC trial that showed the same effect of the SSC4 region on host response to PRRS. Despite the presence of a different B QTL haplotype, the rs340943904 SNP was 100 % concordant with the rs80800372 SNP in 58 commercial Yorkshire boars. Since these boars represent a different genetic background from the ones used in this study, this further supports that rs340943904 is the causal mutation for the SSC4 PRRS host response QTL.

The GBP proteins are excellent candidates to play important roles in host response to bacterial and viral infections. Recent studies of the *GBP5* knockout mouse indicate that GBP5 functions in host defense, inflammasome assembly, and inflammatory response [7]. GBP5 serves as an important adaptor and mediator protein in NLRP3 inflammasome activation. Furthermore, deletion of the 67 C-terminal amino acids of GBP5 abolished the NLRP3 mediated inflammasome assembly and downstream immune response. Protein modeling was used to confirm that the structure of the C-terminus of GBP5 appears to be conserved across the human, mouse and pig [22] (Additional file 16). The splice mutation described in this study results in severely reduced expression of wild-type GBP5 and a frameshift that leads to an early stop codon and truncation of the 88 C-terminal amino acids. These results indicate that pigs with the AA QTL genotype likely have very little to no GBP5 mediated NLRP3 inflammasome response to infection. Relatively little is known about the role of GBP5 in human or animal immune responses to viruses. One study indicates that *GBP5* expression is upregulated during Epstien-Barr viral infection [8]; however, the role of GBP5 in viral immune response is unknown. The implication that GBP5 plays a role in host genetic response to PRRS may indicate additional biological roles for GBP5 in host suppression of viral replication. Therefore, this study may direct new avenues of research into the role of GBP5 in viral immune response and in identification of novel therapeutic targets.

## Conclusions

In conclusion, we present multiple lines of evidence that the introduction of a new splice acceptor site in *GBP5* at the rs340943904 SNP is a putative causal mutation for the SSC4 host PRRS response QTL. First, RNA-seq analyses in two independent populations confirm that *GBP5* was DE as a function of SSC4 QTL genotype. Second, all *GBP5* SNPs in perfect LD with the SSC4 QTL exhibited ASE, with a shift towards higher expression of alleles that were in phase with the favorable B QTL allele. Third, differential splicing of *GBP5* was associated with specific SSC4 QTL genotypes. Fourth, the rs340943904 SNP was validated to be in perfect LD with the SSC4 QTL in an independent genetic background. Fifth, the rs340943904 SNP introduced five nucleotides into exon 10 of the *GBP5* transcript, which is predicted to shift the reading frame and result in an early stop codon that truncates the 88 C-terminal amino acids. Finally, almost no wild-type *GBP5* transcript was produced in individuals with the AA QTL genotype. Since GBP5 is known to play a role in host immune response and inflammation based on studies in the knockout mouse, it is a strong candidate gene for the SSC4 PRRS host response QTL. For these reasons, we believe that the rs340943904 *GBP5* SNP is the causal mutation for this economically important QTL.

## Methods

### Experimental design and population

Sixteen barrows from 8 litters, one AB and one AA individual at the rs80800372 SNP from each litter, were selected from a population of 200 commercial crossbred piglets that had been experimentally infected with PRRS isolate NVSL97-7985 as part of the PRRS Host Genetics Consortium (PHGC trial 3 described in Boddicker et al. [1]). No BB animals were used in the RNA-seq analysis due to the low frequency of this genotype and evidence that the effect of the B allele on host response is dominant [21]. Animals were between 18–28 days of age at day 0 of the trial. Whole blood and serum samples from 0, 4, 7, 10, and 14 dpi were obtained from each individual. Whole blood samples were collected using the Tempus^TM^ Blood RNA Tubes (Life Technologies, Carlsbad, CA, USA) to facilitate RNA isolation and serum samples were used to quantify viral level using a semi-quantitative TaqMan PCR assay [19].

### Ethics statement

All animal experiments were approved by the Kansas State University Institutional Animal Care and use committee under registration number 3000.

### RNA-seq sample pre-processing

Total RNA was isolated from 77 available PHGC3 Tempus^TM^ preserved blood samples using the Tempus^TM^ Spin RNA Isolation Kit (catalogue #: 4380204; Life Technologies, Grand Island, NY, USA) according to the manufacturer’s protocol as previously described [23]. A ND-1000 spectrophotometer (Nano-Drop Technologies, Wilmington, DE, USA) was used to quantify RNA concentrations. The globin transcripts (*HBA* and *HBB*) were reduced using an RNase H based globin reduction method [23] that used porcine specific oligonucleotides modified from an Affymetrix GeneChip GR Protocol for reduction of human globin mRNA. To determine the quality of the RNA both prior to and following globin reduction, RNA samples were assayed for their 28S to 18S rRNA ratio using an Agilent 2100 Bioanalyzer (Agilent Technologies, Inc., Santa Clara, CA, USA). RNA integrity numbers (RIN) were determined using the Bioanalyzer both pre and post globin reduction.

### Library preparation and RNA-sequencing details

Library construction was conducted at the Iowa State University DNA facility with the TruSeq^TM^ library kit (Illumina, Inc., San Diego, USA) according to the manufacturer’s protocol. Sequencing was done on an Illumina HiSeq machine using 100 cycles and the paired-end read methodology to generate 100 base pair reads, as described by the manufacturer (Illumina, Inc., San Diego, USA). Samples from each pair of littermates were allocated to one lane, for a total of 8 lanes, such that litter effects were confounded with lane effects and, thus, power to detect genotype effects was maximized. Initial processing of reads from the HiSeq machine used the Illumina CASAVA (v1.8) software.

### Bioinformatics

#### Read quality assessment

All reads for all 77 samples were analyzed with the Sickle software [24] to remove poor quality sequence and ensure no adaptor remnants remained with any read. Each sample was then checked with FASTQC software (available at: http://www.bioinformatics.babraham.ac.uk/projects/fastqc/) to determine if it had adequate read quality for downstream analyses (i.e. reasonable read length, GC content, low percentage of repeated sequence). Seven of the 77 samples were removed from all further analyses due to poor read mapping (fewer than 1 million reads mapped to the reference genome), or inconsistent sequence-based genotype for the rs80800372 SNP with the SNP chip based genotype for that animal. A total of 70 samples were thus retained for DE analysis (see Additional file 3).

### De novo transcript creation

In order to create the transcripts for the SSC4 QTL region, several approaches were utilized. Reads from all time points from one AB genotype animal were combined and trimmed using Sickle [24] to reduce noise and increase the quality of reads. Then Velvet [25] and Oases [26] software programs were used to create a de novo assembly of transcripts, which were compared back against the existing transcript information for the reference genome assembly (Sscrofa10.2). Transcripts located within the QTL region were identified within the de novo assembly using the BLAST/ BLAT alignment tool at Ensembl (http://uswest.ensembl.org/Sus_scrofa/blastview). All transcripts were translated and compared against the orthologous human protein to validate the accuracy of the transcripts. When full length transcripts were not identified in the de novo transcript assembly, the complete pig transcript was assembled using the appropriate reads that created the full length protein that exhibited high similarity to the corresponding human orthologue based on the BLAST results.

### RNA-seq read processing

To produce a common set of transcript coordinates for all samples, sequence reads for each sample were mapped to the Sscrofa 10.2 reference using Tophat/Bowtie2 [27, 28]. Alignment files from all samples were merged into a single BAM alignment file and Cufflinks [29] was used to define a set of common transcript coordinates for the 70 samples. For each sample, any reads that aligned to the *HBB* and *HBA* reference sequences were removed before htseq analysis [30], using the common transcript coordinates to determine discrete counts for each transcript.

### Variant identification

SNPs in the QTL region were used to perform allele-specific expression analysis. Each BAM file produced by Tophat was reformatted using Picard tools [31] and then used to detect SNPs and small insertion and deletion (INDEL) mutations using the Genome Analysis Tool Kit [32] guided by the Sscrofa build 10.2 genome assembly. To facilitate ASE analyses, the emit_all_bases of GATK was used to capture the read counts at each base, regardless of the observation of an alternative allele. In addition, the down sampling option was turned off to allow for all read counts to be recorded for a SNP variant. These two steps were important to evaluate SNP concordance with genotype at the rs80800372 SNP. Two novel variants identified in GBP5 in this study have been submitted to NCBI dbSNP: ss1414376806 and ss1414376807. Additional SNPs submitted to dbSNP are listed in Additional files 14 and 15.

### RNA-seq 3′ read skewness calculation

Reads from the RNA-seq data were observed to have a 3′ read mapping bias, presumably due to globin depletion and other systematic RNA processing factors [23]. The 3′ read skewness was calculated for each transcript as the proportion of total reads with a midpoint right of the transcript midpoint. The read midpoint was determined using the CIGAR string in each RNA-seq SAM file.

### Differential gene expression analysis

Since the objective of this study was to identify potential causal variants for the SSC4 QTL that impact host response to PRRS, we focused on analysis of the differential expression only of genes within the SSC4 QTL region [1, 2].

### Statistical analysis of RNA-seq gene expression

The trimmed mean of M values (TMM) normalization procedure [33] from the edgeR package in R [34] was used to normalize transcript counts based on the full set of genome-wide counts. This procedure also adjusts for the variation in library size seen across samples. Normalized counts were then log2 transformed to obtain the resulting scaled values used for analysis. A repeated measures linear model was used that included the effects of genotype (AA and AB), day (5 dpi), and genotype-by-day interactions as class variables and the log normalized transcript expression as the response. Additionally and independently for each transcript, a class variable for family and covariates for pre- and post-globin-reduction RIN, and 5′-3′ transcript read skewness were considered to be included based on model selection using Aikake information criterion (AIC) comparisons to select the best model. This process was repeated for 4 error model types: uncorrelated errors, autoregressive order 1(AR[1]) based on day, heterogeneous AR(1) (ARH[1]) based on day, or an unstructured error model. The overall best fitting model was then determined by AIC, separately for each transcript. Contrasts were constructed to estimate and test expression differences between genotypes within and across days. The models were developed in R using the gls function from the nlme package. Multiple testing correction was conducted using the Benjamini & Hochberg FDR [35].

### Quality control and filtering of SNPs for the ASE analysis

The ASE analysis focused on SNP variants within the QTL region. After variant calling, a shell script was developed to extract the genotype and read counts for each sample and each SNP for downstream analysis. Within each individual, reference and alternate allele counts for each SNP were obtained by summing across that individual’s multiple time point samples. Using a count cutoff of at least 2 times the number of time points available for that individual, alleles at a SNP were called as either present or absent in that individual for a given SNP. SNPs were then filtered to compare the behavior of transcripts in LD (i.e. haplotypes) with either the A or B QTL allele. For the initial ASE analyses, SNPs were required to be in perfect LD with the rs80800372 SNP (i.e. the QTL). A total of 64 SNPs in LD with the QTL were analyzed after applying these filters. In addition, a second ASE analysis was conducted that included all heterozygous SNPs regardless of LD status with the QTL. These 67 SNPs were selected using the same count cutoff; however, AA QTL genotyped individuals were included.

### ASE statistical analysis

Two separate analyses of ASE were conducted. The objective of the first ASE analysis was to determine if the alleles of SNPs in LD with the QTL had coordinated expression patterns during PRRS viral infection. Only SNPs in AB QTL genotyped individuals in LD with the QTL were used in this analysis. A mixed nonlinear logistic regression model was fit to the reference and alternate allele counts for each SNP independently. Reference alleles were defined based on the nucleotide present in the Sscrofa 10.2 genome build whereas differing nucleotides were defined as alternate alleles. This model fit a random effect for each individual, fixed effects for the day post infection and possibly included RIN (both prior to and post globin depletion) numeric values as continuous predictors based on model selection using AIC. For each SNP, an estimate of the probability of observing an alternate allele (p_alt_), was obtained and a Wald test was performed to determine whether p_alt_ differed from 0.5. Multiple testing correction was conducted using the Benjamini & Hochberg FDR method [35]. Analyses were performed using the lme4 package in R. The objective of the second ASE analysis was to conducted an exhaustive characterization of ASE in the QTL region. All SNPs not in LD with rs80800372 were analyzed using the same statistical approaches.

### AATI differential splicing analysis

We used AATI capillary electrophoresis (Advanced Analytical, Ames, IA, USA) methodology to validate differential splicing of the *GBP5* transcripts. Two PCR primers were designed to amplify a portion of the *GBP5* transcript from the 3′ end of exon 9 to the 5′ end of exon 10 using PHGC3 blood RNAs. The exon 9 primer consisted of the sequence: 5′- AGATCTGGC TCTCACACAAAGG -3′. The exon 10 primer consisted of the sequence 5′- GCCTCT GCTTGCAAACGTG -3′. This AATI assay identified three alternatively spliced transcripts: wild-type (i.e. capable of producing a functional protein), +5 bps (inserts five base pairs at the start of exon 10), retained intron (transcript includes all of intron 9 between exons 9 and 10). The AATI used internal standards to quantify all alternate transcript peaks at the molarity level. Statistical analysis of the splicing data was conducted using PROC Mixed of SAS. A model was fit to determine if splicing of *GBP5*, measured as the molarity of each alternate transcript, differed with QTL genotype. The model included the fixed effects for genotype (AA or AB), assay date (the AATI batch effect of samples run together on the same day with 4 levels), alternate transcript abundance (the *GBP5* alternate-splicing transcripts: wild-type, +5 bps, and retained intron), and a random effect of animal to account for common effects of individuals across days and shared effects on alternate transcripts.

### Analysis of differential splicing of *GBP5* from RNA-seq data

A script was developed to count the number of reads that uniquely represented the three splice variants of *GBP5* for all time points in the RNA-seq data. These read counts were then analyzed using the same model as described above for the AATI data to determine if differential splicing of the three alternatively spliced transcripts was observed in the RNA-seq data, with the exception that assay date was not necessary in the model.

### Determination of concordance of the rs80800372 variant with rs340943904 in *GBP5* in an independent population

Allele specific PCR protocols were developed to genotype both the rs80800372 and the *GBP5* T > G SNP in intron 9 at position 139,413,978 bps (rs340943904) as described [36]. The rs80800372 SNP was genotyped using a common forward primer: 5′-CCTTCTAGCTTCTCAGTGGA-3′ and specific primers for the “A” QTL allele: reverse primer: 5′-TTCGCTTCTCTAGCCCATTTATGT-3′ or the “B” QTL allele: reverse primer: 5′-TTCGCTTCTCTAGCCCATTTATGC-3′. PCR was conducted with an annealing temperature of 63.5 °C. The T > G SNP in intron 9 at position 139,413,978 bps in *GBP5* was genotyped using a common forward primer: 5′-AGCAAGAACAGCAAAGATGT-3′ and specific primers for the G allele (corresponding to the “A” QTL allele): reverse primer: 5′-TGCAAACGTGCCTCTGAAC-3′ or the A allele (corresponding the “B” QTL allele): reverse primer: 5′-TGCAAACGTGCCTCTGAAA-3′. The PCR annealing temperature was also on 63.5 °C. A total of 58 commercial Yorkshire boars from an independent, validation population were genotyped for these two SNPs to determine the concordance of the *GBP5* rs340943904 SNP with the rs80800372 SNP.

### Validation of RNA-seq results using an independent population

An additional subset of animals from trial 5 of the PHGC [2] was obtained for an independent RNA-seq analysis. The RNA-seq data was created, processed, checked for quality control, and mapped in similar manner as described above and analyzed for differential expression, splicing and ASE. A total of 15 AA and 5 AB genotyped samples were analyzed for differential expression at 0, 4, 7, 11, and 14 dpi. All statistical analysis were conducted with data from the same time points corresponding to the initial PHGC trial 3 study using the same methodology to contrast differences in gene expression by QTL genotype. Read splicing as measured in the RNA-seq data and ASE were also evaluated using the same statistical models as described above for the PHGC trial 3 data using 5 AB and 5 AA across dpi that met the previously mentioned quality control standards.

### Availability of supporting data

The data sets supporting the results in this article are available at NCBI. New variants identified for the ASE analysis of *GBP5* were deposited at dbSNP under accession numbers: ss1414376806 and ss1414376807. All additional variants identified in this study are under the accession numbers listed in Additional files 14 and 15.

## Additional files

Additional file 1:
**A schematic of all genes present in the** ***Sus scrofa*** **chromosome 4 (SSC4) quantitative trait locus (QTL) region for PRRS host response from the Sscrofa 10.2 genome build as presented at Ensembl (acquired July 2, 2014 at:** **http://useast.ensembl.org** **).**
Additional file 2:
**This is a file including the reassembled FASTA sequence of all genes within the** ***Sus scrofa*** **chromosome 4 PRRS host response QTL region at 139136697–140420778 Mbs.**
Additional file 3:
**The number of individuals available at each time point for RNAseq differential expression analysis following read quality and count filtering (N = 70).**
Additional file 4:
**Read mapping statistics for all samples used in the RNAseq analysis.** A total of 77 samples were initially processed, of which, only 70 passed quality control based on read mapping and base level quality scores. Additional file 5:
**Model-estimated log2 fold changes for genes analyzed for differential expression in the Sus scrofa chromosome (SSC) 4 QTL region (139-140 Mb).**
Additional file 6:
**P-values and Benjamini and Hochberg False Discovery Rate (FDR) values associated with model-estimated fold changes in Additional file** 7 **.**
Additional file 7:
**Normalized, model adjusted gene expression and log2 fold changes for all genes tested for differential expression (DE).**
Additional file 8:
**Estimates of allelic proportions and p-values for all SNPs in phase with the rs80800372 SNP B allele tested for allele specific expression within the SSC4 QTL region in AB QTL genotyped individuals.** Results are presented based on averages across all 5 days post infection (dpi) time points. Additional file 9:
**Allele-specific expression analysis of SNPs not in linkage disequlibrium with rs80800372. Results are presented based on averages across all 5 days post infection (dpi) time points.**
Additional file 10:
**Schematic of the guanylate binding protein 5(** ***GBP5*** **)gene and alternate transcripts.** A. A schematic of the *GBP5* gene, including the context of all introns, start and stop codons, coding SNPs, SNPs exhibiting allele specific expression and the putative causal variant causing a new splice site in intron 9 (rs340943904). B. The three alternate *GBP5* transcripts, including the changes in exon 10 and introduction of a premature stop codon that occurs in the translation of two of the alternate transcripts. Intron lengths are based on those used in the *Sus scrofa* build 10.2 genome assembly, with the length of intron 10 determined from sequencing a clone containing the 3′ end of the gene. Additional file 11:
**An image of RNAseq reads mapping to the** ***Sus scrofa*** **10.2 genome build in the integrated genome browser (1) that shows the difference between the three** ***GBP5*** **alternate transcripts: wild-type, +5 bps and retained intron.** This figure presents an individual with the AB QTL genotype. Additional file 12:
**The de novo FASTA nucleotide sequence of the guanylate binding protein 5 transcript.** Exons are shown in alternating colors with the stop and start codons displayed in bold text. Exon coordinates are as follows: Exon 1: 1–219, Exon 2: 220–428, Exon 3: 429–556, Exon 4: 557–666, Exon 5: 667–863, Exon 6: 864–1106, Exon 7: 1107–1387, Exon 8: 1388–1600, Exon 9: 1601–1703, Exon 10: 1704–1885, Exon 11: 1886–2324. Additional file 13:
**A CLUSTALW multiple protein alignment of the AA and AB QTL genotype porcine amino acid sequence compared to bovine, human and mouse GBP5 protein.** Exons are color coded in alternating blue and black colors. The portion of the protein frame-shifted in the AA genotyped individuals is indicated by red text. The full-length amino acid lengths of GBP5 are: pig AA QTL genotype: 498, pig AB QTL (rs80800372) genotype: 586, bovine: 586, human: 586, mouse: 590. The pig AA QTL genotype has 91 amino acids truncated compared to the mouse (88 compared to all other species), which includes portions of exons 10 and 11. Additional file 14:
**Variants in linkage disequilibrium with rs80800372 genotype identified in de novo transcriptome of the Sus scrofa chromosome (SSC) 4 QTL Region.** These variants were discovered and filtered the same as those in Additional file 14: Table S1. All nonsynonymous causing variants that are in LD with the rs80800372 genotype were determined to be minimally deleterious. Additional file 15:
**Variants not in linkage disequilibrium with rs80800372 genotype identified in the de novo transcriptome of the SSC4 QTL Region.**
Additional file 16:
**The protein structure of guanylate binding protein (GBP) 5 appears to be largely conserved between the human, mouse and pig based on comparative protein structure modeling.** A. Human GBP5 modeled using the protein structure of human GBP1 in the SWISS_MODEL protein homology modeler. The C-terminus (C) and N-terminus (N) are marked for reference. B. Mouse GBP5 protein modeled using the human GBP1 protein structure template. C. The Pig B form of the GBP5 protein modeled using the human GBP1 protein structure template. D. The Pig A form of the GBP5 protein modeled using the human GBP1 protein structure template. Note, the A form of the protein is missing the 88 C-terminal amino acids due to a frameshift caused by the insertion of five base pairs just prior to the beginning of exon 10.

## Abbreviations

ASE: Allele specific expression
BAM: Binary sequence alignment/ map format
cDNA: Complementary deoxyribonucleic acid
DE: Differentially expressed
dpi: days post infection
FASTA: a standardized format for the display of nucleic acids
FDR: False discovery rate
GATK: Genome Analysis Toolkit
GBPs: Guanylate binding proteins
GBP5: Guanylate binding protein 5
INDEL: Insertion or deletion variant
LD: Linkage disequilibrium
LPS: Lipopolysaccharide
p_alt_: model estimated probability of expression of the alternate allele
PRRSV: Porcine Respiratory and Reproductive Syndrome virus
PRDC: Porcine respiratory disease complex
PCVAD: Porcine circovirus-associated disease
QTL: Quantitative trait locus
QTN: Quantitative trait nucleotide
RIN: RNA integrity number
RNA: Ribonucleic acid
SNP: Single nucleotide polymorphism
SSC: *Sus scrofa* chromosome
