# Supplementary figures and images for: Identification of a putative quantitative trait nucleotide in guanylate binding protein 5 for host response to PRRS virus infection
Source: BMC Genomics. 2015 May 28;16(1):412. doi: 10.1186/s12864-015-1635-9 (PMC4446061; doi:10.1186/s12864-015-1635-9)

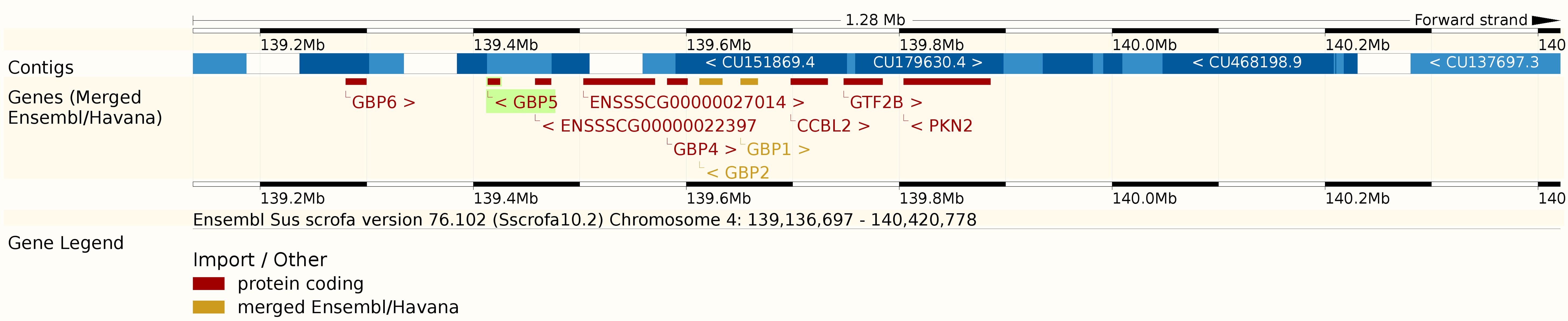

Supplement: Additional file 1: — A schematic of all genes present in the Sus scrofa chromosome 4 (SSC4) quantitative trait locus (QTL) region for PRRS host response from the Sscrofa 10.2 genome build as presented at Ensembl (acquired July 2, 2014 at: http://useast.ensembl.org ). [file 12864_2015_1635_MOESM1_ESM.tiff]

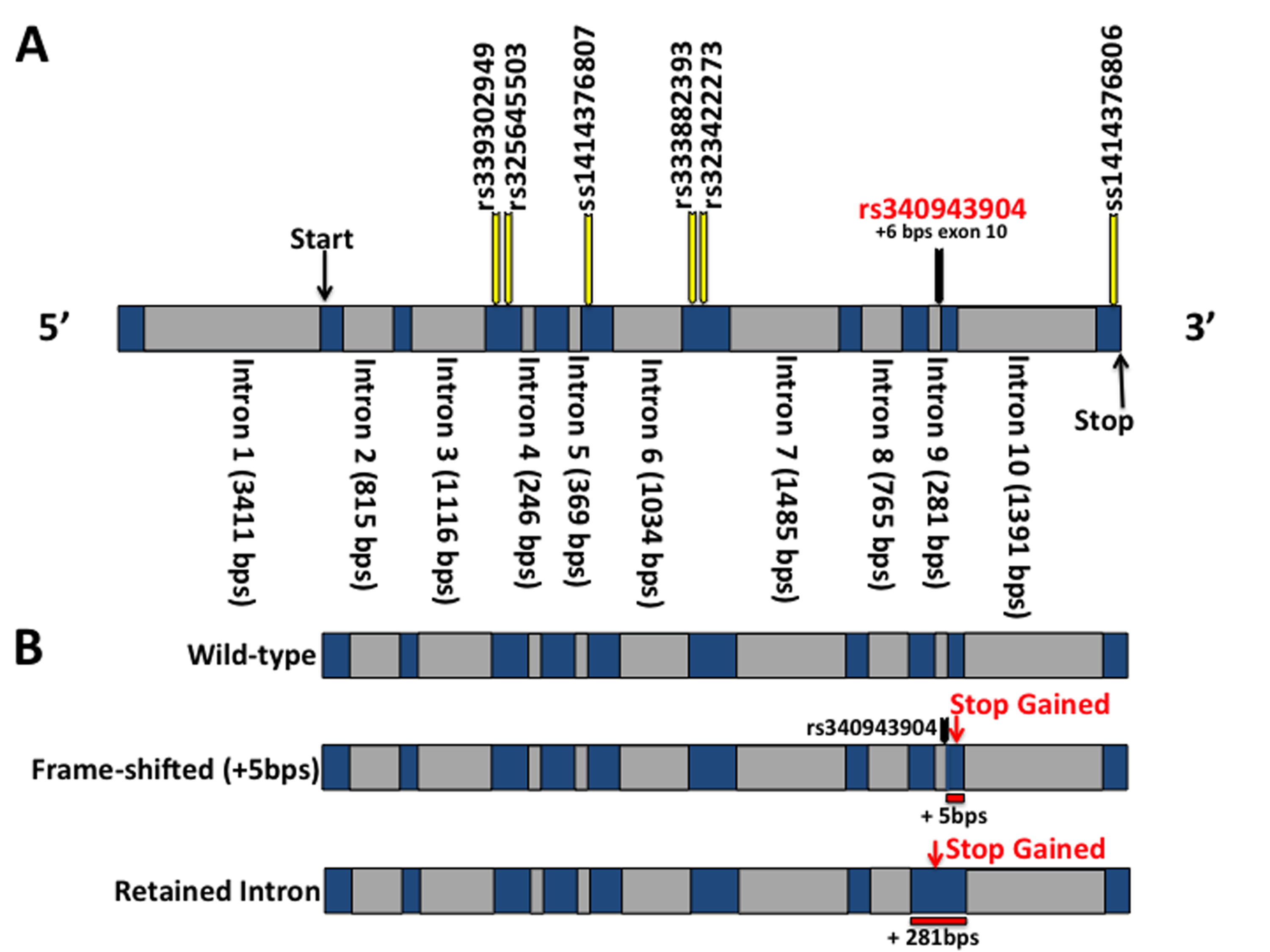

Supplement: Additional file 10: — Schematic of the guanylate binding protein 5( GBP5 )gene and alternate transcripts. A. A schematic of the GBP5 gene, including the context of all introns, start and stop codons, coding SNPs, SNPs exhibiting allele specific expression and the putative causal variant causing a new splice site in intron 9 (rs340943904). B. The three alternate GBP5 transcripts, including the changes in exon 10 and introduction of a premature stop codon that occurs in the translation of two of the alternate transcripts. Intron lengths are based on those used in the Sus scrofa build 10.2 genome assembly, with the length of intron 10 determined from sequencing a clone containing the 3′ end of the gene. [file 12864_2015_1635_MOESM10_ESM.tiff]

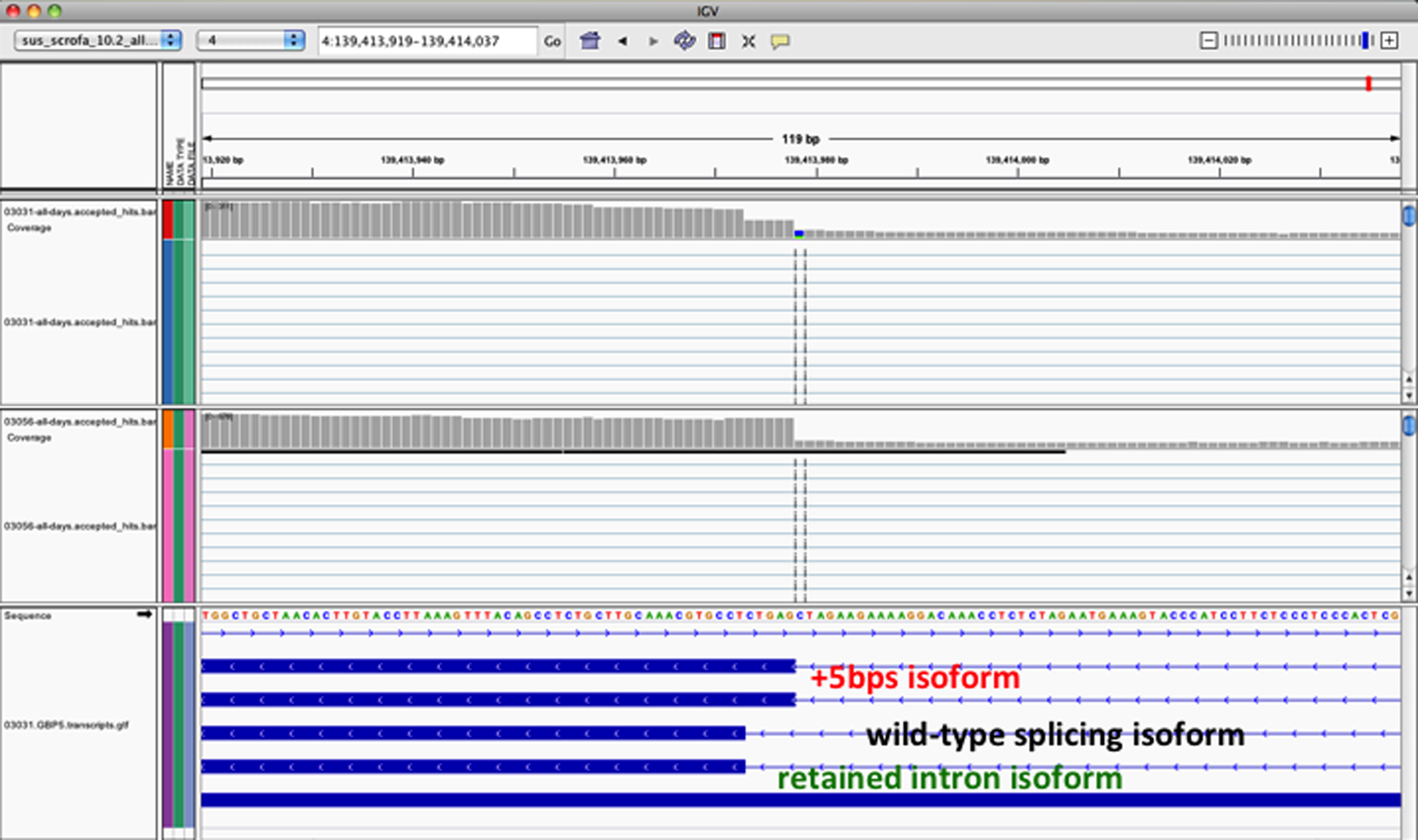

Supplement: Additional file 11: — An image of RNAseq reads mapping to the Sus scrofa 10.2 genome build in the integrated genome browser (1) that shows the difference between the three GBP5 alternate transcripts: wild-type, +5 bps and retained intron. This figure presents an individual with the AB QTL genotype. [file 12864_2015_1635_MOESM11_ESM.tiff]

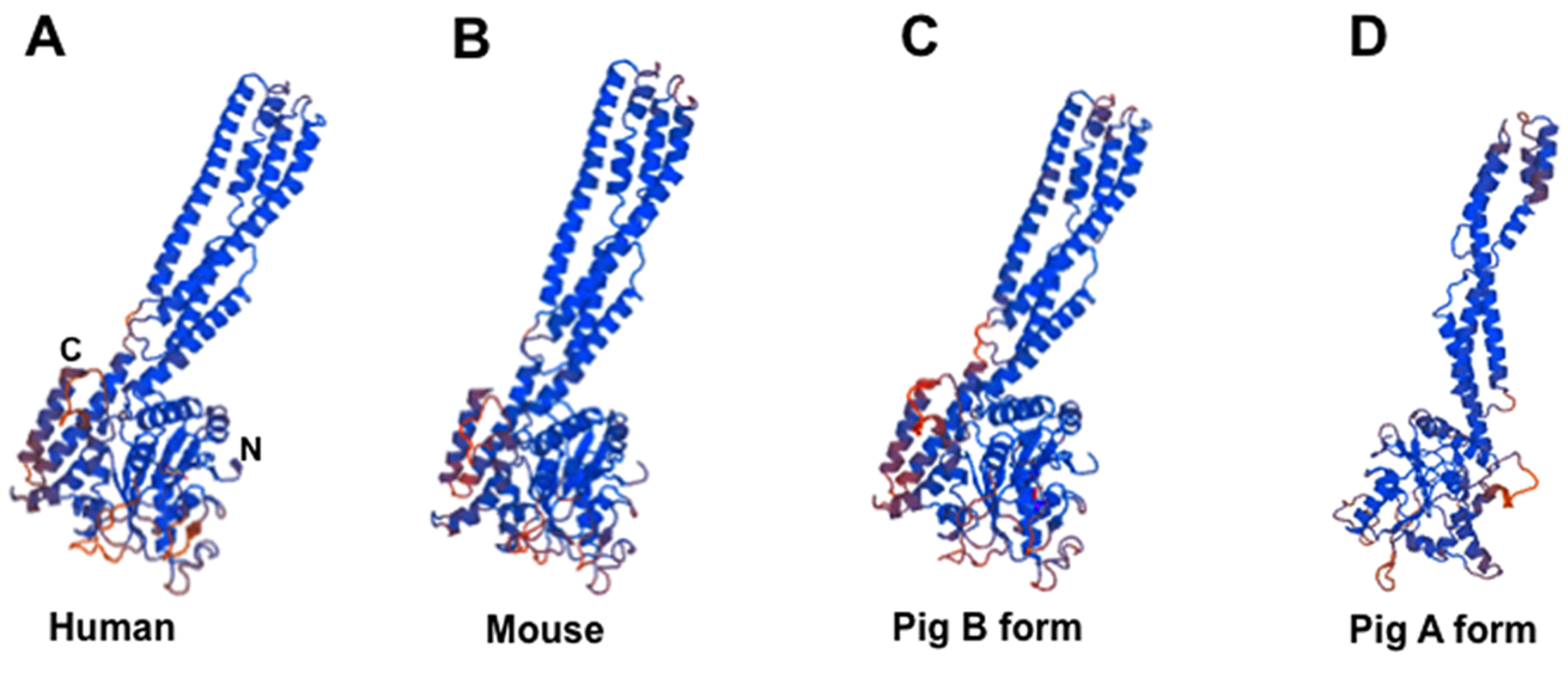

Supplement: Additional file 16: — The protein structure of guanylate binding protein (GBP) 5 appears to be largely conserved between the human, mouse and pig based on comparative protein structure modeling. A. Human GBP5 modeled using the protein structure of human GBP1 in the SWISS_MODEL protein homology modeler. The C-terminus (C) and N-terminus (N) are marked for reference. B. Mouse GBP5 protein modeled using the human GBP1 protein structure template. C. The Pig B form of the GBP5 protein modeled using the human GBP1 protein structure template. D. The Pig A form of the GBP5 protein modeled using the human GBP1 protein structure template. Note, the A form of the protein is missing the 88 C-terminal amino acids due to a frameshift caused by the insertion of five base pairs just prior to the beginning of exon 10. [file 12864_2015_1635_MOESM16_ESM.tiff]
