# Supplementary material for: Identification of a putative quantitative trait nucleotide in guanylate binding protein 5 for host response to PRRS virus infection
Source: BMC Genomics. 2015 May 28;16(1):412. doi: 10.1186/s12864-015-1635-9 (PMC4446061; doi:10.1186/s12864-015-1635-9)
Supplement: Additional file 3: — The number of individuals available at each time point for RNAseq differential expression analysis following read quality and count filtering (N = 70). [file 12864_2015_1635_MOESM3_ESM.docx]

| **rs80800372 genotype** | **DPI 0** | **DPI 4** | **DPI 7** | **DPI 11** | **DPI 14** |
| --- | --- | --- | --- | --- | --- |
| **AA** | 8 | 7 | 8 | 5 | 7 |
| **AB** | 6 | 7 | 7 | 7 | 8 |
