# Supplementary material for: Identification of a putative quantitative trait nucleotide in guanylate binding protein 5 for host response to PRRS virus infection
Source: BMC Genomics. 2015 May 28;16(1):412. doi: 10.1186/s12864-015-1635-9 (PMC4446061; doi:10.1186/s12864-015-1635-9)
Supplement: Additional file 4: — Read mapping statistics for all samples used in the RNAseq analysis. A total of 77 samples were initially processed, of which, only 70 passed quality control based on read mapping and base level quality scores. [file 12864_2015_1635_MOESM4_ESM.docx]

| **Sample*** | **# Reads Mapped** | **% Reads Mapping** | **# Bases Mapped** |
| --- | --- | --- | --- |
| 03031-0DPI | 238979 | 37.07 | 23897900 |
| 03031-4DPI | 19646497 | 85.58 | 1964649700 |
| 03031-7DPI | 35863374 | 84.89 | 3586337400 |
| 03031-10DPI | 19893230 | 83.09 | 1989323000 |
| 03031-14DPI | 25758098 | 81.64 | 2575809800 |
| 03056-0DPI | 46944324 | 84.49 | 4694432400 |
| 03056-4DPI | 53233236 | 83.38 | 5323323600 |
| 03056-7DPI | 33040815 | 85.28 | 3304081500 |
| 03056-14DPI | 23044019 | 87.52 | 2304401900 |
| 03068-0DPI | 24635101 | 80.53 | 2463510100 |
| 03068-4DPI | 34685762 | 80.57 | 3468576200 |
| 03068-7DPI | 24227260 | 83.53 | 2422726000 |
| 03068-10DPI | 21423346 | 83.39 | 2142334600 |
| 03068-14DPI | 57397587 | 83.14 | 5739758700 |
| 03074-4DPI | 18207520 | 73.34 | 1820752000 |
| 03074-7DPI | 22905279 | 80.80 | 2290527900 |
| 03074-10DPI | 22651255 | 82.38 | 2265125500 |
| 03074-14DPI | 40910098 | 84.47 | 4091009800 |
| 03089-0DPI | 41459200 | 83.07 | 4145920000 |
| 03089-4DPI | 51582588 | 82.44 | 5158258800 |
| 03089-7DPI | 15247950 | 84.53 | 1524795000 |
| 03089-10DPI | 4359822 | 47.65 | 435982200 |
| 03089-14DPI | 23209078 | 75.64 | 2320907800 |
| 03094-0DPI | 15424049 | 85.94 | 1542404900 |
| 03094-4DPI | 26681682 | 87.43 | 2668168200 |
| 03094-7DPI | 10288285 | 83.35 | 1028828500 |
| 03094-10DPI | 7941472 | 84.82 | 794147200 |
| 03094-14DPI | 18430060 | 85.89 | 1843006000 |
| 03101-0DPI | 8186467 | 67.58 | 818646700 |
| 03101-4DPI | 53519114 | 82.18 | 5351911400 |
| 03101-7DPI | 25243670 | 85.83 | 2524367000 |
| 03101-10DPI | 33455214 | 86.34 | 3345521400 |
| 03101-14DPI | 93206231 | 85.56 | 9320623100 |
| 03112-0DPI | 13755915 | 85.74 | 1375591500 |
| 03112-4DPI | 26999 | 45.09 | 2699900 |
| 03112-7DPI | 5477779 | 26.11 | 547777900 |
| 03112-10DPI | 42699549 | 85.27 | 4269954900 |
| 03112-14DPI | 102626918 | 83.66 | 10262691800 |
| 03113-0DPI | 45980492 | 82.57 | 4598049200 |
| 03113-4DPI | 36375059 | 80.02 | 3637505900 |
| 03113-7DPI | 9081127 | 68.92 | 908112700 |
| 03113-10DPI | 28993046 | 86.77 | 2899304600 |
| 03113-14DPI | 41221375 | 83.91 | 4122137500 |
| 03122-0DPI | 14651963 | 78.13 | 1465196300 |
| 03122-4DPI | 18966473 | 77.27 | 1896647300 |
| 03122-7DPI | 19383469 | 82.35 | 1938346900 |
| 03122-10DPI | 67790050 | 85.08 | 6779005000 |
| 03122-14DPI | 36542184 | 84.30 | 3654218400 |
| 03129-0DPI | 24634438 | 82.36 | 2463443800 |
| 03129-4DPI | 12866117 | 82.08 | 1286611700 |
| 03129-7DPI | 17051061 | 83.30 | 1705106100 |
| 03129-10DPI | 124161207 | 83.68 | 12416120700 |
| 03148-0DPI | 16514691 | 72.48 | 1651469100 |
| 03148-4DPI | 65359621 | 83.88 | 6535962100 |
| 03148-7DPI | 49546761 | 86.11 | 4954676100 |
| 03148-10DPI | 32118970 | 80.10 | 3211897000 |
| 03148-14DPI | 36748180 | 82.33 | 3674818000 |
| 03159-0DPI | 49804607 | 85.60 | 4980460700 |
| 03159-4DPI | 28634486 | 90.26 | 2863448600 |
| 03159-7DPI | 11734207 | 70.06 | 1173420700 |
| 03159-10DPI | 69111121 | 83.10 | 6911112100 |
| 03159-14DPI | 48837005 | 83.95 | 4883700500 |
| 03170-0DPI | 15345158 | 84.83 | 1534515800 |
| 03170-4DPI | 12150799 | 69.16 | 1215079900 |
| 03170-7DPI | 15487800 | 83.38 | 1548780000 |
| 03170-10DPI | 62862103 | 84.99 | 6286210300 |
| 03170-14DPI | 54407670 | 83.11 | 5440767000 |
| 03174-0DPI | 19869577 | 85.21 | 1986957700 |
| 03174-4DPI | 40192422 | 86.45 | 4019242200 |
| 03174-7DPI | 37167357 | 84.02 | 3716735700 |
| 03174-10DPI | 25519986 | 85.18 | 2551998600 |
| 03174-14DPI | 23177195 | 83.17 | 2317719500 |
| 03192-0DPI | 60805652 | 86.10 | 6080565200 |
| 03192-4DPI | 41892295 | 83.68 | 4189229500 |
| 03192-7DPI | 28414716 | 83.34 | 2841471600 |
| 03192-10DPI | 35095820 | 82.46 | 3509582000 |
| 03192-14DPI | 24497105 | 84.74 | 2449710500 |

^^^Samples in red were removed following quality control.

*Seven samples shown in red were removed for downstream analyses due to low RIN score (1), no or low number or reads mapped to the reference genome (3), or inconsistent sequence-based genotype with previous SNP chip based genotypes (3).
