# Supplementary material for: Identification of a putative quantitative trait nucleotide in guanylate binding protein 5 for host response to PRRS virus infection
Source: BMC Genomics. 2015 May 28;16(1):412. doi: 10.1186/s12864-015-1635-9 (PMC4446061; doi:10.1186/s12864-015-1635-9)
Supplement: Additional file 5: — Model-estimated log2 fold changes for genes analyzed for differential expression in the Sus scrofa chromosome (SSC) 4 QTL region (139-140 Mb). [file 12864_2015_1635_MOESM5_ESM.docx]

|  | **Log2 Fold Change*** | |  |  |  |  |
| --- | --- | --- | --- | --- | --- | --- |
| **Gene** | **Day 0** | **Day 4** | **Day 7** | **Day 10** | **Day 14** | **Main** |
| GBP2 | -1.021769745 | -0.25174875 | 0.299995999 | 0.37656707 | 0.111901003 | -0.097010884 |
| GBP1 | -1.524726825 | -0.385431643 | 0.216944176 | 0.20925938 | 0.409248457 | -0.214941291 |
| GTF2B | -1.933344494 | -0.069702248 | 0.10083514 | -0.031633127 | 0.179845719 | -0.350799802 |
| CCBL2 | -1.605694454 | -0.079409774 | 0.051998498 | 0.248597305 | -0.046263963 | -0.286154478 |
| GBP4 | -1.015379165 | -0.041124911 | 0.198438028 | 0.402884354 | 0.419091691 | -0.007218 |
| GBP5 | -0.493153256 | 0.419363088 | 0.89688496 | 0.937585619 | 0.843526178 | 0.520841318 |
| GBP6 | -0.770258878 | -0.130782921 | 0.580323679 | 0.91152627 | 0.591504744 | 0.236462579 |
| PKN2 | -1.355274859 | -0.013342546 | -0.06436858 | 0.108576426 | 0.065234963 | -0.251834919 |
|  |  |  |  |  |  |  |

*Note, red text indicates significance at p < 0.05.
