# Supplementary material for: Identification of a putative quantitative trait nucleotide in guanylate binding protein 5 for host response to PRRS virus infection
Source: BMC Genomics. 2015 May 28;16(1):412. doi: 10.1186/s12864-015-1635-9 (PMC4446061; doi:10.1186/s12864-015-1635-9)
Supplement: Additional file 6: — P-values and Benjamini and Hochberg False Discovery Rate (FDR) values associated with model-estimated fold changes in Additional file 7 . [file 12864_2015_1635_MOESM6_ESM.docx]

|  | **Pvalues*** |  |  |  |  |  |
| --- | --- | --- | --- | --- | --- | --- |
| **Gene** | **Day 0** | **Day 4** | **Day 7** | **Day 10** | **Day 14** | **Main** |
| GBP2 | 0.329987816 | 0.564383541 | 0.36304011 | 0.077845614 | 0.751232116 | 0.741837333 |
| GBP1 | 0.206128478 | 0.516337517 | 0.453412396 | 0.115189146 | 0.084564314 | 0.542981815 |
| GTF2B | 0.150679195 | 0.813624674 | 0.524062877 | 0.821246783 | 0.264559495 | 0.299761761 |
| CCBL2 | 0.140760374 | 0.67033156 | 0.820729378 | 0.671050198 | 0.712554367 | 0.284654461 |
| GBP4 | 0.367729537 | 0.890937263 | 0.401292158 | 0.004411177 | 0.079165744 | 0.977504448 |
| GBP5 | 0.632579712 | 0.320344949 | 0.000922629 | 0.000297519 | 0.006002756 | 0.051587873 |
| GBP6 | 0.44527365 | 0.752965983 | 0.073474544 | 3.23E-09 | 0.116454843 | 0.309430188 |
| PKN2 | 0.199515767 | 0.927615665 | 0.610821678 | 0.524385084 | 0.606969453 | 0.259739204 |
|  |  |  |  |  |  |  |
|  | **Benjamini & Hochberg FDR** | | | |  |  |
| **Gene** | **Day 0** | **Day 4** | **Day 7** | **Day 10** | **Day 14** | **Main** |
| GBP2 | 0.490306049 | 0.927615665 | 0.698081918 | 0.155691228 | 0.751232116 | 0.847814095 |
| GBP1 | 0.412256955 | 0.927615665 | 0.698081918 | 0.184302634 | 0.225504837 | 0.723975753 |
| GTF2B | 0.412256955 | 0.927615665 | 0.698081918 | 0.821246783 | 0.423295192 | 0.495088301 |
| CCBL2 | 0.412256955 | 0.927615665 | 0.820729378 | 0.766914512 | 0.751232116 | 0.495088301 |
| GBP4 | 0.490306049 | 0.927615665 | 0.698081918 | 0.011763138 | 0.225504837 | 0.977504448 |
| GBP5 | 0.632579712 | 0.927615665 | 0.007381032 | 0.001190074 | 0.048022046 | 0.412702987 |
| GBP6 | 0.508884171 | 0.927615665 | 0.293898175 | 2.58E-08 | 0.232909686 | 0.495088301 |
| PKN2 | 0.412256955 | 0.927615665 | 0.698081918 | 0.699180112 | 0.751232116 | 0.495088301 |

*Note, red text indicates significance at p < 0.05 and the corresponding FDR values.
