# Supplementary material for: Identification of a putative quantitative trait nucleotide in guanylate binding protein 5 for host response to PRRS virus infection
Source: BMC Genomics. 2015 May 28;16(1):412. doi: 10.1186/s12864-015-1635-9 (PMC4446061; doi:10.1186/s12864-015-1635-9)
Supplement: Additional file 8: — Estimates of allelic proportions and p-values for all SNPs in phase with the rs80800372 SNP B allele tested for allele specific expression within the SSC4 QTL region in AB QTL genotyped individuals. Results are presented based on averages across all 5 days post infection (dpi) time points. [file 12864_2015_1635_MOESM8_ESM.docx]

| **NCBI dbSNP ssID** | **Gene** | **Locus^*,ŧ^** | **Prop.in.phase.allele^^^** | **pvalue** | **FDR^+^** |
| --- | --- | --- | --- | --- | --- |
| 1751076281 | CCBL2 | 434 | 0.455 | 1.85E-06 | 3.33E-06 |
| 1751076319 | GBP1 | 283 | 0.507 | 0.354497868 | 0.408084755 |
| 1751076321 | GBP1 | 284 | 0.511 | 0.162171386 | 0.195792283 |
| 1751076323 | GBP1 | 325 | 0.468 | 0.008590518 | 0.012325526 |
| 1751076364 | GBP1 | 824 | 0.530 | 0.045089341 | 0.058734799 |
| 1751076287 | GBP1 | 1205 | 0.524 | 0.076100268 | 0.096588802 |
| 1751076343 | GBP1 | 1493 | 0.546 | 1.81E-05 | 3.14E-05 |
| 1751076290 | GBP1 | 1727 | 0.452 | 0.016311763 | 0.025603274 |
| 1751076292 | GBP1 | 2368 | 0.463 | 4.86E-05 | 9.92E-05 |
| 1751076347 | GBP1 | 2374 | 0.141 | 2.56E-33 | 1.59E-32 |
| 1751076296 | GBP1 | 2408 | 0.484 | 0.065878909 | 0.089769063 |
| 1751076317 | GBP1 | 2438 | 0.510 | 0.207974835 | 0.243291317 |
| 1751076403 | GBP2 | 31 | 0.470 | 6.57E-06 | 1.16E-05 |
| 1751076382 | GBP2 | 66 | 0.476 | 6.29E-05 | 0.000105627 |
| 1751076405 | GBP2 | 449 | 0.493 | 0.464465802 | 0.51665297 |
| 1751076407 | GBP2 | 619 | 0.466 | 9.24E-07 | 1.76E-06 |
| 1751076366 | GBP2 | 624 | 0.468 | 1.43E-06 | 2.68E-06 |
| 1751076395 | GBP2 | 1194 | 0.560 | 7.43E-07 | 1.47E-06 |
| 1751076378 | GBP2 | 1705 | 0.543 | 0.000820105 | 0.001288736 |
| 1751076380 | GBP2 | 1752 | 0.527 | 0.002102266 | 0.003201912 |
| 1751076420 | GBP2 | 1887 | 0.519 | 0.104443091 | 0.13088438 |
| 1751076422 | GBP2 | 1906 | 0.483 | 0.111827832 | 0.136678462 |
| 1751076452 | GBP4 | 46 | 0.566 | 0.011321814 | 0.01601228 |
| 1751076435 | GBP4 | 441 | 0.563 | 1.27E-09 | 3.31E-09 |
| 1751076437 | GBP4 | 578 | 0.600 | 6.25E-11 | 1.77E-10 |
| 1751076447 | GBP4 | 656 | 0.626 | 1.49E-14 | 4.92E-14 |
| 1751076454 | GBP4 | 661 | 0.612 | 1.54E-13 | 4.63E-13 |
| 1751076430 | GBP4 | 1206 | 0.369 | 6.62E-12 | 2.35E-11 |
| 1751076439 | GBP4 | 1682 | 0.595 | 2.10E-12 | 6.13E-12 |
| 1751076433 | GBP4 | 1873 | 0.359 | 4.44E-09 | 1.13E-08 |
| 1751076450 | GBP4 | 1931 | 0.410 | 0.000141131 | 0.000232867 |
| 1751076441 | GBP4 | 1999 | 0.468 | 0.280124758 | 0.330147036 |
| 1751076443 | GBP4 | 2092 | 0.498 | 0.939547744 | 0.949134966 |
| 1751076445 | GBP4 | 2105 | 0.502 | 0.957723575 | 0.957723575 |
| 1751076461 | GBP5 | 595 | 0.741 | 1.65E-24 | 7.08E-24 |
| 1751076479 | GBP5 | 610 | 0.747 | 1.27E-34 | 6.01E-34 |
| 1751076477 | GBP5 | 866 | 0.731 | 9.90E-19 | 3.77E-18 |
| 1751076471 | GBP5 | 1221 | 0.695 | 3.13E-21 | 1.29E-20 |
| 1751076473 | GBP5 | 1230 | 0.688 | 4.21E-18 | 1.54E-17 |
| 1751076475 | GBP5 | 2086 | 0.681 | 4.79E-110 | 4.74E-109 |
| 1751076499 | GBP6 | 106 | 0.459 | 0.028916774 | 0.039760565 |
| 1751076505 | GBP6 | 171 | 0.183 | 4.88E-05 | 9.92E-05 |
| 1751076497 | GBP6 | 671 | 0.668 | 7.05E-11 | 1.94E-10 |
| 1751076481 | GBP6 | 1429 | 0.663 | 1.40E-07 | 2.95E-07 |
| 1751076503 | GBP6 | 1489 | 0.685 | 6.81E-09 | 1.69E-08 |
| 1751076507 | GBP6 | 2322 | 0.341 | 0.000581861 | 0.00106104 |
| 1751076522 | GBP6 | 4293 | 0.228 | 9.34E-14 | 3.86E-13 |
| 1751076533 | GTF2B | 202 | 0.593 | 0.000644759 | 0.001029535 |
| 1751076535 | GTF2B | 392 | 0.448 | 2.70E-07 | 5.57E-07 |
| 1751076539 | GTF2B | 852 | 0.439 | 5.79E-11 | 1.99E-10 |
| 1751076537 | GTF2B | 918 | 0.463 | 5.75E-08 | 1.24E-07 |
| 1751076561 | PKN2 | 2257 | 0.462 | 0.33034903 | 0.384759459 |
| 1751076555 | PKN2 | 2437 | 0.490 | 0.799388632 | 0.824369527 |
| 1751076541 | PKN2 | 2438 | 0.495 | 0.876795903 | 0.894874169 |
| 1751076557 | PKN2 | 2622 | 0.515 | 0.626350668 | 0.666832677 |
| 1751076546 | PKN2 | 2907 | 0.600 | 7.70E-07 | 1.49E-06 |
| 1751076548 | PKN2 | 2943 | 0.582 | 0.000176676 | 0.000286737 |
| 1751076559 | PKN2 | 3125 | 0.563 | 0.189182242 | 0.225651108 |
| 1751076563 | PKN2 | 3428 | 0.513 | 0.626418576 | 0.666832677 |
| 1751076551 | PKN2 | 3455 | 0.516 | 0.457988026 | 0.515236529 |

*Locus refers to the base position in the transcript relative to the 5’ end of the transcript.

**^ŧ^**SNPs in alternate phase with rs80800372 (the SSC4 PRRS host response QTL) include: GBP4:578, GBP4:656, GBP4:661, GBP4:1682, GBP5:2086, GBP6:1429, GBP6:1489, GTF2B:918, PKN2:3455. In addition these SNPs were excluded due to quality control filtering prior to analysis: GBP6:3006, GBP6:3822.

**^^^**Proportion of alleles for each SNP in phase with the rs80800372 B allele, averaged across all dpi time points.

**^+^**FDR=False Discovery Rate.
