# Supplementary material for: Identification of a putative quantitative trait nucleotide in guanylate binding protein 5 for host response to PRRS virus infection
Source: BMC Genomics. 2015 May 28;16(1):412. doi: 10.1186/s12864-015-1635-9 (PMC4446061; doi:10.1186/s12864-015-1635-9)
Supplement: Additional file 9: — Allele-specific expression analysis of SNPs not in linkage disequlibrium with rs80800372. Results are presented based on averages across all 5 days post infection (dpi) time points. [file 12864_2015_1635_MOESM9_ESM.docx]

| **NCBI dbSNP ssID** | **Gene** | **Locus*** | **Alt.allele.prop^^^** | **pvalue** | **FDR^+^** |
| --- | --- | --- | --- | --- | --- |
| 1751076299 | GBP1 | 270 | 0.542 | 1.17E-06 | 2.70E-06 |
| 1751076301 | GBP1 | 271 | 0.537 | 2.13E-05 | 4.64E-05 |
| 1751076307 | GBP1 | 679 | 0.094 | 3.04E-151 | 6.28E-150 |
| 1751076328 | GBP1 | 680 | 0.100 | 1.09E-151 | 2.71E-150 |
| 1751076330 | GBP1 | 684 | 0.099 | 1.85E-194 | 1.15E-192 |
| 1751076309 | GBP1 | 1185 | 0.507 | 0.354497868 | 0.408084755 |
| 1751076354 | GBP1 | 1188 | 0.511 | 0.162171386 | 0.195792283 |
| 1751076332 | GBP1 | 1189 | 0.468 | 0.008590518 | 0.012325526 |
| 1751076334 | GBP1 | 1192 | 0.530 | 0.045089341 | 0.058734799 |
| 1751076336 | GBP1 | 1193 | 0.524 | 0.076100268 | 0.096588802 |
| 1751076338 | GBP1 | 1195 | 0.546 | 1.81E-05 | 3.14E-05 |
| 1751076311 | GBP1 | 1196 | 0.602 | 1.04E-13 | 4.16E-13 |
| 1751076356 | GBP1 | 1197 | 0.247 | 2.44E-113 | 3.36E-112 |
| 1751076341 | GBP1 | 1198 | 0.513 | 0.204978892 | 0.243291317 |
| 1751076313 | GBP1 | 1199 | 0.470 | 6.57E-06 | 0.000011608 |
| 1751076349 | GBP1 | 2375 | 0.498 | 0.951521174 | 0.951521174 |
| 1751076294 | GBP1 | 2380 | 0.469 | 0.245319264 | 0.284295222 |
| 1751076315 | GBP1 | 2382 | 0.484 | 0.550903315 | 0.578915348 |
| 1751076358 | GBP1 | 2715 | 0.560 | 7.43E-07 | 1.47E-06 |
| 1751076303 | GBP1 | 2716 | 0.547 | 0.169380717 | 0.212153626 |
| 1751076305 | GBP1 | 2718 | 0.543 | 0.000820105 | 0.001288736 |
| 1751076360 | GBP1 | 2720 | 0.363 | 0.037093715 | 0.053483961 |
| 1751076351 | GBP1 | 2721 | 0.370 | 0.046897646 | 0.065340541 |
| 1751076362 | GBP1 | 2726 | 0.483 | 0.111827832 | 0.136678462 |
| 1751076424 | GBP2 | 3 | 0.563 | 1.27E-09 | 3.31E-09 |
| 1751076426 | GBP2 | 463 | 0.400 | 6.25E-11 | 1.77E-10 |
| 1751076409 | GBP2 | 686 | 0.374 | 1.49E-14 | 4.92E-14 |
| 1751076385 | GBP2 | 688 | 0.388 | 1.54E-13 | 4.63E-13 |
| 1751076368 | GBP2 | 690 | 0.405 | 2.10E-12 | 6.13E-12 |
| 1751076411 | GBP2 | 691 | 0.359 | 4.44E-09 | 1.13E-08 |
| 1751076387 | GBP2 | 692 | 0.410 | 0.000141131 | 0.000232867 |
| 1751076370 | GBP2 | 693 | 0.468 | 0.280124758 | 0.330147036 |
| 1751076389 | GBP2 | 695 | 0.502 | 0.957723575 | 0.957723575 |
| 1751076428 | GBP2 | 700 | 0.747 | 1.27E-34 | 6.01E-34 |
| 1751076391 | GBP2 | 764 | 0.731 | 9.90E-19 | 3.77E-18 |
| 1751076393 | GBP2 | 837 | 0.103 | 5.02E-169 | 1.56E-167 |
| 1751076413 | GBP2 | 839 | 0.319 | 4.79E-110 | 4.74E-109 |
| 1751076372 | GBP2 | 840 | 0.459 | 0.028916774 | 0.039760565 |
| 1751076397 | GBP2 | 1537 | 0.315 | 6.81E-09 | 1.69E-08 |
| 1751076415 | GBP2 | 1555 | 0.189 | 5.08E-26 | 2.86E-25 |
| 1751076374 | GBP2 | 1559 | 0.236 | 1.75E-08 | 5.05E-08 |
| 1751076376 | GBP2 | 1561 | 0.593 | 0.000644759 | 0.001029535 |
| 1751076417 | GBP2 | 1565 | 0.448 | 2.70E-07 | 5.57E-07 |
| 1751076469 | GBP5 | 824 | 0.551 | 6.66E-19 | 3.06E-18 |
| 1751076524 | GBP6 | 121 | 0.606 | 1.33E-12 | 4.99E-12 |
| 1751076526 | GBP6 | 165 | 0.063 | 8.91E-07 | 2.08E-06 |
| 1751076528 | GBP6 | 202 | 0.537 | 5.75E-08 | 1.24E-07 |
| 1751076530 | GBP6 | 205 | 0.462 | 0.33034903 | 0.384759459 |
| 1751076486 | GBP6 | 209 | 0.008 | 0.077003274 | 0.102671032 |
| 1751076516 | GBP6 | 850 | 0.300 | 3.22E-10 | 1.08E-09 |
| 1751076501 | GBP6 | 1222 | 0.261 | 3.42E-19 | 1.70E-18 |
| 1751076518 | GBP6 | 1284 | 0.279 | 4.50E-07 | 1.07E-06 |
| 1751076488 | GBP6 | 4564 | 0.415 | 0.127068488 | 0.165857816 |
| 1751076490 | GBP6 | 4587 | 0.443 | 0.318071099 | 0.361842352 |

*Locus refers to the base position in the transcript relative to the 5’ end of the transcript. In addition these SNPs were excluded due to quality control filtering prior to analysis: GBP1:509, GBP2:1576, GBP2:1600, GBP5:225, GBP6:1637, GBP6:4626, GBP6:4627.

**^^^**Proportion of alternate alleles averaged across all dpi time points.

**^+^**FDR=False Discovery Rate.
