# Supplementary material for: Identification of a putative quantitative trait nucleotide in guanylate binding protein 5 for host response to PRRS virus infection
Source: BMC Genomics. 2015 May 28;16(1):412. doi: 10.1186/s12864-015-1635-9 (PMC4446061; doi:10.1186/s12864-015-1635-9)
Supplement: Additional file 12: — The de novo FASTA nucleotide sequence of the guanylate binding protein 5 transcript. Exons are shown in alternating colors with the stop and start codons displayed in bold text. Exon coordinates are as follows: Exon 1: 1–219, Exon 2: 220–428, Exon 3: 429–556, Exon 4: 557–666, Exon 5: 667–863, Exon 6: 864–1106, Exon 7: 1107–1387, Exon 8: 1388–1600, Exon 9: 1601–1703, Exon 10: 1704–1885, Exon 11: 1886–2324. [file 12864_2015_1635_MOESM12_ESM.docx]

**GBP5 transcript (exons are shown in alternating colors)**

CTTCCACTTTTGGTTTTGTTTTGTATTCTAATCCTCTGCCTTGTGGAAGGCAGCTCAGTGGAGAGAAAGGAAATTCCCAGGACAGGCTTTTTTTTTCTTTAAAGTATATCAGAAAAACACAGCTAAACTAAGGGACAGCGTTACAGGTCTCACCAAGTTGTCATGAGGCAGCTTCCTCCCTTGGTCTAAAAGATCTCTGCTACTGACCTTACAGTTAAGATCAGAGGACACCCTAGAC**ATG**GCCTCAGGGGTGCACATGCCCGAACCACAGTGCCTCATTGAGAACATCAATGGGCGACTGGCGGTGAACCCGAAAGCGCTGAAGCTCCTGTCTGCCATCAAGCAGCCCCTGGTGGTGGTGGCTATCGTGGGCCTGTACCGCACAGGCAAATCCTACCTGATGAACAAGCTGGCTGGGAAGAACAAGGGCTTCTCTGTGGGCTCCACGGTGCAGTCTCACACAAAGGGCATCTGGATGTGGTGCGTGCCTCACCCCAGGAAGCCAGACCACACCCTGGTCCTTCTGGACACCGAGGGACTCGGGGATGTGGAGAAGGGCGACAAGAAAAATGACACCCAGATCTTTGTGCTGGCACTGTTACTGAGCAGTACCTTTGTATACAACACCATGAACAAAATTGACCAGAGGGCAATCGACCTCTTGCACTACGTGGCAGAACTAGCCACATGGCTCCAAACAGTATCCTCAACTGATGCTGACGAGGTGTCGGGTCCAGAGGACTCTGTGAGCAACTGTCCAGACTTAGTGTGGACTCTGAGAGATTTCTTCCTTGACCTGGAAGTAAATGGACACCCCATCACAACAGATGAATATCTGGAGAATTCGCTGAGGCCAAAGCCAGGGGCTGATAAAAGTCTTCAGAATTTTAATTTGCCCCGTCAGTGTATACAGAAATTCTTTCCCACAAAGAAATGCTTTATTTTTGACTCCCCCACTCATCGGAAGAAGCTTGCCCAGCTTGAGACGCTACATGATGATGACTTGGAACCGGACTTTGTACAACAAGTGGCGGAATTCTGTTCCTACATCTTCAGCCACTCCAAATCTAAAACTCTTCCAGAAGGCAGCAAGGCCAATGGGTCCCATCTAGAGAGAGTGGTGCTGACCTACGTGAAAGCCATCAGCAGTGGGGACCTGCCCTGTGTGGAGAACACAGTCCTGGCCTTGGCGCAGGTTAAGAACTCGGCTGCAATGAAAACGGCCATTGCCCACTATGACCAGCTGATGGGCCAGAATCTGCACCTGCCCACAGAGACCCTGCAGGAGCTGCTGGACCTGCACAGGATCTGTAAGAAAGTGGCCATCGAAGTATTCGTAATGAATTCTTTCAAGGATGTGGACCACGGGTTCCAGAAAAAATTAGAGACCCTGCTAGAAGCCAAACAGAATGAACTTCATGAAAGGAACTTGAAGACATCACTAGACCGTTGCTCATCTTTACTTCAGGTTATTTTTGAACCTCTAGAAGAAGAAGTGAAACAGGGGTTTTATTCAATACCAGGGGGGCATCGTCTCTTCATGCAGAGGAGAGAAGAGCTGAAGGCAGTGTACTATCAGGTGCCCTGGAAAGGGCTACAGGCTGAAGAAGCTCTGCGGAAATATTTACAGTCCAAGGAGTCTATGAATGTTACGATTTTCCAGACAGATCTGGCTCTCACACAAAGGGAGAAGGAGATGGAAGAGGCACGTTTGCAAGCAGAGGCTGTAAACTTTAAGGTACAAGTGTTAGCAGCCATCCTAACACAGCAACACCAAATGATGGAGCAGCGGCAAAGATTCTATCAGGAACAAGTGAGACGAATGGAAATAAACAGACTGCACCAGCAGGTATTGCAACAGAGAGCCCAGGAACGTTATCTCCAGGAAGAAGCTAAAAGGATCCAGGAGAGAGCCCAAGCTGAGAACAAGAGACTTCAAGATGAGCTCGAGCATCTGCAGATAAATGACTCGAATGATGATAAATGTATCATACTT**TAA**AGAGCTAAACACAAAAACTTCCCTTTCTTGTTCACTTTCCCTGAAGACACAAGGGAACACGAAACTGTAAAACCTGAGACAATCACCATTTGAATAAACTTCACAATAATTATATTGAACTTACATGTGGAATCTAAAAAAAGGATGCAATGAACCTCTTTGCAGAACAGATACTGACTCACAAACTTTGAAAAACTTACGGTTGCCAAATGAGACAGGTTTGGGGATGGGGGGATGTGCTGGGGGTTTGAGATGGAAATGCTATGATTTGGTTGTGATGATCACTGTACAACTATAAATGTAATAAAATTCATTCAGTAATGAC

**Exon 1:** 1-219

**Exon 2:** 220-428

**Exon 3:** 429-556

**Exon 4:** 557-666

**Exon 5:** 667-863

**Exon 6:** 864-1106

**Exon 7:** 1107-1387

**Exon 8:** 1388-1600

**Exon 9:** 1601-1703

**Exon 10:** 1704-1885

**Exon 11:** 1886-2324
