# Supplementary material for: Identification of a putative quantitative trait nucleotide in guanylate binding protein 5 for host response to PRRS virus infection
Source: BMC Genomics. 2015 May 28;16(1):412. doi: 10.1186/s12864-015-1635-9 (PMC4446061; doi:10.1186/s12864-015-1635-9)
Supplement: Additional file 13: — A CLUSTALW multiple protein alignment of the AA and AB QTL genotype porcine amino acid sequence compared to bovine, human and mouse GBP5 protein. Exons are color coded in alternating blue and black colors. The portion of the protein frame-shifted in the AA genotyped individuals is indicated by red text. The full-length amino acid lengths of GBP5 are: pig AA QTL genotype: 498, pig AB QTL (rs80800372) genotype: 586, bovine: 586, human: 586, mouse: 590. The pig AA QTL genotype has 91 amino acids truncated compared to the mouse (88 compared to all other species), which includes portions of exons 10 and 11. [file 12864_2015_1635_MOESM13_ESM.docx]

**Pig_AA --MASGVHMPEPQCLIENINGRLAVNPKALKLLSAIKQPLVVVAIVGLYRTGKSYLMNKL**

**Pig_AB --MASGVHMPEPQCLIENINGRLAVNPKALKLLSAIKQPLVVVAIVGLYRTGKSYLMNKL**

**Bovine MDMAPLSHMPEPLCLIENTNGPLLVNPEALKILSAIRQPVVVVAIMGLYRTGKSYLMNKL**

**Human --MALEIHMSDPMCLIENFNEQLKVNQEALEILSAITQPVVVVAIVGLYRTGKSYLMNKL**

**Mouse --MAPEIHMPEPLCLIGSTEGHLVTNQEALKILSAITQPVVVVAIVGLYRTGKSYLMNKL**

**** **.:* *** . : * .* :**::**** **:*****:****************

**Pig_AA AGKNKGFSVGSTVQSHTKGIWMWCVPHPRKPDHTLVLLDTEGLGDVEKGDKKNDTQIFVL**

**Pig_AB AGKNKGFSVGSTVQSHTKGIWMWCVPHPRKPDHTLVLLDTEGLGDVEKGDKKNDTQIFVL**

**Bovine AGKNKGFSVGSTVQSHTKGIWMWCVPHPKKPNHTLVLLDTEGLGDVEKGDKTNDSHISVL**

**Human AGKNKGFSVASTVQSHTKGIWIWCVPHPNWPNHTLVLLDTEGLGDVEKADNKNDIQIFAL**

**Mouse AGKEKGFSVGSTVQSHTKGIWMWCVPHPQKPDHTLVLLDTEGLGDVEKDDKKNDTQIFAL**

*****:*****.***********:******. *:**************** *:.** :* .***

**Pig_AA ALLLSSTFVYNTMNKIDQRAIDLLHYVAELATWLQTVSSTDADEVSGPE-DSVSNCPDLV**

**Pig_AB ALLLSSTFVYNTMNKIDQRAIDLLHYVAELATWLQTVSSTDADEVSGPE-DSVSNCPDLV**

**Bovine ALLLCSTFVYNTMNKIDQGAIDLLHYVTELSHLLRIDTSPNLNRVDDAA-DFVRICPNLV**

**Human ALLLSSTFVYNTVNKIDQGAIDLLHNVTELTDLLKARNSPDLDRVEDPA-DSASFFPDLV**

**Mouse AILLSSTFVYNTMNKIDQGAIDLLHNVTELTDLLRTRNSSDSNQTEGEGPADMSFFPDLV**

***:**.*******:***** ****** *:**: *: .*.: :.... *:****

**Pig_AA WTLRDFFLDLEVNGHPITTDEYLENSLRPKPGADKSLQNFNLPRQCIQKFFPTKKCFIFD**

**Pig_AB WTLRDFFLDLEVNGHPITTDEYLENSLRPKPGADKSLQNFNLPRQCIQKFFPTKKCFIFD**

**Bovine WTLRDFYLGLEANGQLITADEYLENSLRPKQGTNQHLQNFNLPRLCIQKFFPVKKCFIFD**

**Human WTLRDFCLGLEIDGQLVTPDEYLENSLRPKQGSDQRVQNFNLPRLCIQKFFPKKKCFIFD**

**Mouse WTLRDFFLDLQANGHAITSDEYLENSLKLKQGSDERTQTFNLPRLCIQKFFPVKKCFVFD**

******** *.*: :*: :*.********: * *::: *.***** ******* ****:****

**Pig_AA SPTHRKKLAQLETLHDDDLEPDFVQQVAEFCSYIFSHSKSKTLPEGSKANGSHLERVVLT**

**Pig_AB SPTHRKKLAQLETLHDDDLEPDFVQQVAEFCSYIFSHSKSKTLPEGSKANGSHLERVVLT**

**Bovine LPTHQKKLAQLETLHNDDLDPKFVQQVAEFCSYIFSHSKTKTLSGGIKASGSHLENLVQT**

**Human LPAHQKKLAQLETLPDDELEPEFVQQVTEFCSYIFSHSMTKTLPGGIMVNGSRLKNLVLT**

**Mouse APALGSKLSQLPTLSNEELNSDFVQDLSEFCSHIFTQSKTKTLPGGIQVNGPRLESLVLT**

***: .**:** ** :::*:..***:::****:**::* :***. * ..*.:*: :* ***

**Pig_AA YVKAISSGDLPCVENTVLALAQVKNSAAMKTAIAHYDQLMGQNLHLPTETLQELLDLHRI**

**Pig_AB YVKAISSGDLPCVENTVLALAQVKNSAAMKTAIAHYDQLMGQNLHLPTETLQELLDLHRI**

**Bovine YVNAINSGDLPCMESEVLTLAQIKNLAAVQKAIAHYDQKMGQKLQLPTETFQELLDLHRA**

**Human YVNAISSGDLPCIENAVLALAQRENSAAVQKAIAHYDQQMGQKVQLPMETLQELLDLHRT**

**Mouse YVDAINSGALPSIENTVVTLARRENSAAVQKAIGHYDQLMSEKVQLPTETLQELLDLHRT**

****.**.** **.:*. *::**: :* **::.**.**** *.::::** **:**********

**Pig_AA CKKVAIEVFVMNSFKDVDHGFQKKLETLLEAKQNELHERNLKTSLDRCSSLLQVIFEPLE**

**Pig_AB CKKVAIEVFVMNSFKDVDHGFQKKLETLLEAKQNELHERNLKTSLDRCSSLLQVIFEPLE**

**Bovine SEKEAIEVFMKNSFKDEDQGYQKKLEMQLAAKQNDFHERNLEASQNRCSALLQDIFHPLE**

**Human SEREAIEVFMKNSFKDVDQSFQKELETLLDAKQNDICKRNLEASSDYCSALLKDIFGPLE**

**Mouse CEREAIEIFRKHSFKDEGEFFQKELESLLSAKQDEICKKNADASAALCSTLLGSIFKPLE**

**.:: ***:* :**** .. :**:** * ***::: ::* .:* **:** ** *****

**Pig_AA EEVKQGFYSIPGGHRLFMQRREELKAVYYQVPWKGLQAEEALRKYLQSKESMNVTIFQTD**

**Pig_AB EEVKQGFYSIPGGHRLFMQRREELKAVYYQVPWKGLQAEEALRKYLQSKESMNVTIFQTD**

**Bovine ENVKQGVYSKPGGHCLFIQQRDELKAKYNQEPRKGIQAEEALQKYLESKESMSVTILQTD**

**Human EAVKQGIYSKPGGHNLFIQKTEELKAKYYREPRKGIQAEEVLQKYLKSKESVSHAILQTD**

**Mouse QEVAQEFYHKPGGHKLFLQRMEQLKANYRQQPGKGTQAEEVLQTYLNAKETVSRTILQTD**

**: * * .* **** **:*: ::*** * : * ** ****.*:.**::**::. :*:*****

**Pig_AA LALTQREKEMEAQRHVCKQRL---------------------------------------**

**Pig_AB LALTQREKEMEEARLQAEAVNFKVQVLAAILTQQHQMMEQRQRFYQEQVRRMEINRLHQQ**

**Bovine LALTAMEKEMEGITEEFIKAEAQR--LMESLMQHQQMMESRKIFHQEQVRLMETNRVYQE**

**Human QALTETEKKKKEAQVKAEAEKAEAQRLAAIQRQNEQMMQERERLHQEQVRQMEIAKQNWL**

**Mouse QVLTDKEIQSKAEQERAEAARLEAQRLEAIRIQEEQRKAEMERQHQEQLRQIALEKARVA**

**.** * : :**

**Pig_AA ----------------------------------------------------**

**Pig_AB VLQQRAQERYLQEEAKRIQERAQAENKRLQDELEHLQINDSNDDKCIIL---**

**Bovine ALQQRAMERQLQEEVKKLKEKLQAENRKLHDELQNLLRNDSPDDTCLLL---**

**Human AEQQKMQEQQMQEQAAQLSTTFQAQNRSLLSELQHAQRTVNNDDPCVLL---**

**Mouse QEQQWILKQRAQEEADRIKAEQEAQLRALQQQLQHMREMNHHRRHHHDCVIS**
