# Supplementary material for: Identification of a putative quantitative trait nucleotide in guanylate binding protein 5 for host response to PRRS virus infection
Source: BMC Genomics. 2015 May 28;16(1):412. doi: 10.1186/s12864-015-1635-9 (PMC4446061; doi:10.1186/s12864-015-1635-9)
Supplement: Additional file 14: — Variants in linkage disequilibrium with rs80800372 genotype identified in de novo transcriptome of the Sus scrofa chromosome (SSC) 4 QTL Region. These variants were discovered and filtered the same as those in Additional file 14: Table S1. All nonsynonymous causing variants that are in LD with the rs80800372 genotype were determined to be minimally deleterious. [file 12864_2015_1635_MOESM14_ESM.docx]

| **NCBI dbSNP ssID** | **Gene Name** | **Variant ID^^^** | **Variant Location & consequence** |
| --- | --- | --- | --- |
| 1751076319 | GBP1 | C.283.A | NonSynonymous: P to H |
| 1751076321 | GBP1 | C.284.A | Synonymous |
| 1751076323 | GBP1 | C.325.T | NonSynonymous: A to V |
| 1751076364 | GBP1 | T.824.A | NonSynonymous: N to K |
| 1751076287 | GBP1 | A.1205.G | 3’ UTR |
| 1751076343 | GBP1 | G.1493.A | 3’ UTR |
| 1751076290 | GBP1 | A.1727.T | 3’ UTR |
| 1751076292 | GBP1 | A.2368.G | 3’ UTR |
| 1751076347 | GBP1 | G.2374.A | 3’ UTR |
| 1751076296 | GBP1 | A.2408.G | 3’ UTR |
| 1751076317 | GBP1 | C.2438.T | 3’ UTR |
| 1751076403 | GBP2 | G.31.A | 5’UTR |
| 1751076382 | GBP2 | C.66.G | 5’ UTR |
| 1751076405 | GBP2 | G.449.T | 5’ UTR |
| 1751076407 | GBP2 | G.619.A | 5’ UTR |
| 1751076366 | GBP2 | A.624.G | 5’ UTR |
| 1751076395 | GBP2 | G.1194.C | 5’ UTR |
| 1751076378 | GBP2 | C.1705.A | 5’ UTR |
| 1751076380 | GBP2 | C.1752.G | 5’ UTR |
| 1751076420 | GBP2 | T.1887.A | 5’ UTR |
| 1751076422 | GBP2 | T.1906.C | 5’ UTR |
| 1751076452 | GBP4 | T.46.C | 5’ UTR |
| 1751076435 | GBP4 | A.441.G | NonSynonymous: H to R |
| 1751076437 | GBP4 | A.578.G | NonSynonymous: T to A |
| 1751076447 | GBP4 | G.656.C | NonSynonymous: E to Q |
| 1751076454 | GBP4 | T.661.C | Synonymous |
| 1751076430 | GBP4 | A.1206.T | NonSynonymous: E to V |
| 1751076439 | GBP4 | C.1682.T | 3’ UTR |
| 1751076433 | GBP4 | A.1873.G | 3’ UTR |
| 1751076450 | GBP4 | T.1931.C | 3’ UTR |
| 1751076441 | GBP4 | C.1999.T | 3’ UTR |
| 1751076443 | GBP4 | G.2092.T | 3’ UTR |
| 1751076445 | GBP4 | G.2105.A | 3’ UTR |
| 1751076281 | CCBL2 | T.434.A | 5’ UTR |
| 1751076461 | GBP5 | A.595.C | Synonymous |
| 1751076479 | GBP5 | T.610.C | Synonymous |
| 1751076477 | GBP5 | G.866.A | NonSynonymous: A to T |
| 1751076471 | GBP5 | C.1221.A | NonSynonymous: T to K |
| 1751076473 | GBP5 | C.1230.T | NonSynonymous: A to V |
| 1751076475 | GBP5 | C.2086.T | 3’ UTR |
| 1751076499 | GBP6 | C.106.T | 5’ UTR |
| 1751076505 | GBP6 | C.171.T | 5’ UTR |
| 1751076497 | GBP6 | A.671.G | NonSynonymous: M to V |
| 1751076481 | GBP6 | A.1429.G | Synonymous |
| 1751076503 | GBP6 | C.1489.T | Synonymous |
| 1751076507 | GBP6 | C.2322.T | 3’ UTR |
| 1751076509 | GBP6 | C.2344.T | 3’ UTR |
| 1751076511 | GBP6 | C.3006.T | 3’ UTR |
| 1751076520 | GBP6 | G.3822.T | 3’ UTR |
| 1751076522 | GBP6 | G.4293.T | 3’ UTR |
| 1751076533 | GTF2B | A.202.G | 5’ UTR |
| 1751076535 | GTF2B | A.392.G | 5’ UTR |
| 1751076539 | GTF2B | G.852.A | 5’ UTR |
| 1751076537 | GTF2B | A.918.C | 5’ UTR |
| 1751076561 | PKN2 | T.2257.C | 3’ UTR |
| 1751076555 | PKN2 | G.2437.A | 3’ UTR |
| 1751076541 | PKN2 | A.2438.T | 3’ UTR |
| 1751076557 | PKN2 | G.2622.A | 3’ UTR |
| 1751076546 | PKN2 | C.2907.T | 3’ UTR |
| 1751076548 | PKN2 | C.2943.T | 3’ UTR |
| 1751076559 | PKN2 | G.3125.A | 3’ UTR |
| 1751076563 | PKN2 | T.3428.C | 3’ UTR |
| 1751076551 | PKN2 | C.3455.G | 3’ UTR |

These variants were discovered and filtered the same as those in Table1. All nonsynonymous causing variants that are in LD with the rs80800372 genotype were determined to be minimally deleterious.

^^^Variant names include the reference allele, the position starting at the 5’ end in the de novo transcript found in supplemental file 1, followed by the alternate allele.
