# Supplementary material for: Identification of a putative quantitative trait nucleotide in guanylate binding protein 5 for host response to PRRS virus infection
Source: BMC Genomics. 2015 May 28;16(1):412. doi: 10.1186/s12864-015-1635-9 (PMC4446061; doi:10.1186/s12864-015-1635-9)
Supplement: Additional file 15: — Variants not in linkage disequilibrium with rs80800372 genotype identified in the de novo transcriptome of the SSC4 QTL Region. [file 12864_2015_1635_MOESM15_ESM.docx]

| **NCBI dbSNP ssID** | **Gene Name** | **Variant ID^^^** | **Variant Location & consequence** |
| --- | --- | --- | --- |
| 1751076299 | GBP1 | A.270.G | NonSynonymous: K to E |
| 1751076301 | GBP1 | A.271.G | NonSynonyous: K to R |
| 1751076325 | GBP1 | C.509.G | Synonymous |
| 1751076307 | GBP1 | A.679.C | NonSynonymous: D to A |
| 1751076328 | GBP1 | C.680.T | Synonymous |
| 1751076330 | GBP1 | C.684.G | NonSynonymous: L to V |
| 1751076309 | GBP1 | C.1185.T | NonSynonymous: P to S |
| 1751076354 | GBP1 | T.1188.A | Stop Lost^+^ |
| 1751076332 | GBP1 | G.1189.A | Synonymous |
| 1751076334 | GBP1 | G.1192.T | 3’ UTR |
| 1751076336 | GBP1 | G.1193.T | 3’ UTR |
| 1751076338 | GBP1 | G.1195.A | 3’ UTR |
| 1751076311 | GBP1 | C.1196.T | 3’ UTR |
| 1751076356 | GBP1 | T.1197.G | 3’ UTR |
| 1751076341 | GBP1 | G.1198.A | 3’ UTR |
| 1751076313 | GBP1 | C.1199.G | 3’ UTR |
| 1751076349 | GBP1 | G.2375.A | 3’ UTR |
| 1751076294 | GBP1 | A.2380.C | 3’ UTR |
| 1751076315 | GBP1 | C.2382.A | 3’ UTR |
| 1751076358 | GBP1 | T.2715.G | 3’ UTR |
| 1751076303 | GBP1 | A.2716.G | 3’ UTR |
| 1751076305 | GBP1 | A.2718.T | 3’ UTR |
| 1751076360 | GBP1 | T.2720.G | 3’ UTR |
| 1751076351 | GBP1 | G.2721.C | 3’ UTR |
| 1751076362 | GBP1 | T.2726.G | 3’ UTR |
| 1751076424 | GBP2 | T.3.C | 5’ UTR |
| 1751076426 | GBP2 | T.463.C | 5’ UTR |
| 1751076409 | GBP2 | G.686.A | 5’ UTR |
| 1751076385 | GBP2 | C.688.T | 5’ UTR |
| 1751076368 | GBP2 | A.690.T | 5’ UTR |
| 1751076411 | GBP2 | G.691.T | 5’ UTR |
| 1751076387 | GBP2 | C.692.G | 5’ UTR |
| 1751076370 | GBP2 | A.693.T | 5’ UTR |
| 1751076389 | GBP2 | C.695.T | 5’ UTR |
| 1751076428 | GBP2 | T.700.G | 5’ UTR |
| 1751076391 | GBP2 | C.764.T | 5’ UTR |
| 1751076393 | GBP2 | C.837.G | 5’ UTR |
| 1751076413 | GBP2 | G.839.T | 5’ UTR |
| 1751076372 | GBP2 | A.840.C | 5’ UTR |
| 1751076397 | GBP2 | G.1537.A | 5’ UTR |
| 1751076415 | GBP2 | T.1555.G | 5’ UTR |
| 1751076374 | GBP2 | C.1559.G | 5’ UTR |
| 1751076376 | GBP2 | C.1561.A | 5’ UTR |
| 1751076417 | GBP2 | T.1565.C | 5’ UTR |
| 1751076399 | GBP2 | G.1576.C | 5’ UTR |
| 1751076401 | GBP2 | G.1600.C | 5’ UTR |
| 1751076456 | GBP5 | A.225.G | 5’ UTR |
| 1751076469 | GBP5 | A.824.G | NonSynonymous: T to A |
| 1751076524 | GBP6 | T.121.C | 5’ UTR |
| 1751076526 | GBP6 | T.165.C | 5’ UTR |
| 1751076528 | GBP6 | T.202.C | Synonymous |
| 1751076530 | GBP6 | T.205.C | Synonymous |
| 1751076486 | GBP6 | A.209.G | NonSynonymous: T to A |
| 1751076516 | GBP6 | C.850.T | Synonymous |
| 1751076501 | GBP6 | C.1222.T | Synonymous |
| 1751076518 | GBP6 | G.1284.T | NonSynonymous: R to L |
| 1751076484 | GBP6 | A.1637.G | NonSynonymous: R to G |
| 1751076488 | GBP6 | A.4564.G | 3’ UTR |
| 1751076490 | GBP6 | A.4587.G | 3’ UTR |
| 1751076513 | GBP6 | C.4626.T | 3’ UTR |
| 1751076494 | GBP6 | A.4627.G | 3’ UTR |
| 1751076553 | PKN2 | C.6153.T | 3’ UTR |
| 1751076544 | PKN2 | A.6154.G | 3’ UTR |

These variants were identified by aligning the samples against the de novo transcriptome using Bowtie2 and GATK to call variants. A filter requiring a depth of 20x across all samples, 25% or more of samples containing the variant, and a combined sample score of >200, was then applied.

^^^Variant names include the reference allele, the position starting at the 5’ end in the de novo transcript found in supplemental file 1, followed by the alternate allele.

^+^This variant appears to create a protein with an extra 212 amino acids.
